# Comparative analyses of lipidomes and transcriptomes reveal a concerted action of multiple defensive systems against photooxidative stress in *Haematococcus pluvialis*

**DOI:** 10.1093/jxb/eru206

**Published:** 2014-05-12

**Authors:** Yunho Gwak, Yong-sic Hwang, Baobei Wang, Minju Kim, Jooyeon Jeong, Choul-Gyun Lee, Qiang Hu, Danxiang Han, EonSeon Jin

**Affiliations:** ^1^Department of Life Science, Research Institute for Natural Science, Hanyang University, Seoul, 133-791, South Korea; ^2^Department of Bioscience and Biotechnology, Konkuk University, Seoul, 143-701, South Korea; ^3^College of Technology and Innovation, Arizona State University, Mesa, AZ 85212, USA; ^4^Department of Biotechnology, Institute of Industrial Biotechnology, Inha University, Incheon, 402-751, South Korea; ^5^Center for Microalgal Biotechnology and Biofuels, Institute of Hydrobiology, Chinese Academy of Sciences, Wuhan, Hubei 430072, China

**Keywords:** Astaxanthin, high irradiance, lipidome, photooxidative stress, transcriptome, triacylglycerol.

## Abstract

*Haematococcus pluvialis* cells predominantly remain in the macrozooid stage under favourable environmental conditions but are rapidly differentiated into haematocysts upon exposure to various environmental stresses. Haematocysts are characterized by massive accumulations of astaxanthin sequestered in cytosolic oil globules. Lipidomic analyses revealed that synthesis of the storage lipid triacylglycerol (TAG) was substantially stimulated under high irradiance. Simultaneously, remodelling of membrane glycerolipids occurred as a result of dramatic reductions in chloroplast membrane glycolipids but remained unchanged or declined slightly in extraplastidic membrane glycerolipids. *De novo* assembly of transcriptomes revealed the genomic and metabolic features of this unsequenced microalga. Comparative transcriptomic analysis showed that so-called resting cells (haematocysts) may be more active than fast-growing vegetative cells (macrozooids) regarding metabolic pathways and functions. Comparative transcriptomic analyses of astaxanthin biosynthesis suggested that the non-mevalonate pathway mediated the synthesis of isopentenyl diphosphate, as the majority of genes involved in subsequent astaxanthin biosynthesis were substantially up-regulated under high irradiance, with the genes encoding phytoene synthase, phytoene desaturase, and β-carotene hydroxylase identified as the most prominent regulatory components. Accumulation of TAG under high irradiance was attributed to moderate up-regulation of *de novo* fatty acid biosynthesis at the gene level as well as to moderate elevation of the TAG assembly pathways. Additionally, inferred from transcriptomic differentiation, an increase in reactive oxygen species (ROS) scavenging activity, a decrease in ROS production, and the relaxation of over-reduction of the photosynthetic electron transport chain will work together to protect against photooxidative stress in *H. pluvialis* under high irradiance.

## Summary

This study represents the first genome-wide transcriptomic and lipidomic analysis of *H. pluvialis* to reveal the regulation of astaxanthin biosynthesis and lipid metabolism during the encystment process under high irradiation.

## Introduction

Ever since it was first described by J. von Flotow in 1844, *Haematococcus pluvialis* (Chlorophyceae, order Vovlocales), a unicellular freshwater microalga, has drawn significant scientific attention due to its potential as a natural resource of the high-value carotenoid astaxanthin (Lorenz and Cysewski, 2000; Cardozo *et al.*, 2007; Leonardi *et al.*, 2011). Astaxanthin is one of the most powerful natural antioxidants and is a natural food colourant. Therefore, it is of great commercial importance in the nutraceutical and aquaculture industries (Lorenz and Cysewski, 2000). In *H. pluvialis* cells, astaxanthin is present in the form of fatty acyl mono- or diesters under various environmental stress conditions such as nutrient deprivation, high irradiation, and high salt concentrations. Upon exposure to these stressors, the green motile flagellated and non-motile palmella forms of *H. pluvialis* cells rapidly transform into resting cells called haematocysts or aplanospores. This change is accompanied by massive accumulation of astaxanthin in cytosolic lipid bodies, turning the cells a distinct red colour (Boussiba, 2000; Grunewald *et al.*, 2001; Steinbrenner and Linden, 2003; Damiani *et al.*, 2010). The haematocysts can accumulate this versatile secondary carotenoid at up to 4% of cellular dry weight (DW) which is two orders of magnitude greater than that found in yeasts and higher plants (Boussiba, 2000; Lorenz and Cysewski, 2000; Steinbrenner and Linden, 2001; Wang *et al.*, 2009*a*).

Astaxanthin accumulation is generally assumed to be a survival strategy adopted by some microalgae grown in stressful environments, acting as a sunscreen against excessive irradiance (Hagen *et al.*, 1994), an antioxidant preventing production of reactive oxygen species (ROS) (Kobayashi *et al.*, 1997; Li *et al.*, 2008), and a carbon or energy reserve (Quin *et al.*, 2008) (for a review, see Lemoine and Schoefs, 2010). As a 40-carbon isoprenoid compound, astaxanthin is synthesized from two five-carbon isoprene precursors, isopentenyl diphosphate (IPP) and dimethylallyl diphosphate (DMAPP). Sequential addition of three IPP molecules to DMAPP yields 20-carbon geranylgeranyl pyrophosphate (GGPP), two molecules of which are condensed head-to-tail to tetraterpene phytoene, the precursor for all carotenoids. The genes encoding a suite of enzymes that catalyse the formation of astaxanthin from phytoene have been cloned and characterized in *H. pluvialis* (Steinbrenner and Linden, 2003; Steinbrenner and Sandmann, 2006; Vidhyavathi *et al.*, 2008); however, the genetic details of the biosynthetic route(s) leading to the formation of IPP and DMAPP have yet to be elucidated. Two unrelated IPP biosynthesis pathways—the mevalonate (MVA) pathway and the 2-C-methyl-d-erythritol 4-phosphate (MEP) pathway—have been described in vascular plants. Although it has been suggested that the MEP pathway is responsible for carotenoid biosynthesis in vascular plants and eukaryotic algae, direct evidence pointing to its role in astaxanthin biosynthesis in *H. pluvialis* is lacking at the gene level (Sun *et al.*, 1998; Grunewald *et al.*, 2000; Hagen and Grunewald, 2000). A genome-wide survey, along with differential gene expression analysis, would be necessary to identify all genes involved in astaxanthin biosynthesis in this organism. A comparison of their expression profiles would then be required in order to facilitate identification of key regulatory genes.

The accumulation of astaxanthin has been correlated with increasing fatty acid biosynthesis in *H. pluvialis* cells under stress conditions (Zhekisheva *et al.*, 2002). In haematocysts, the fatty acid content can be as high as 30% of DW and comprised of >80% unsaturated fatty acids, making this organism a promising strain for biodiesel production (Zhekisheva *et al.*, 2002; Miao *et al.*, 2008; Damiani *et al.*, 2010; Peled *et al.*, 2011; Lei *et al.*, 2012). Under stress conditions, a large portion of *de novo* synthesized fatty acids are assembled into triacylglycerols (TAGs), which are co-sequestered along with astaxanthin esters into cytosolic lipid bodies through an unknown mechanism. Thus, unravelling the metabolic regulation of TAG biosynthesis may enable further elucidation of astaxanthin synthesis and deposition in *H. pluvialis*, which may in turn guide rational physiological or genetic manipulation to improve astaxanthin and lipid production in this organism. In the current study, a lipidomics approach was employed to analyse TAG molecular species and membrane glycerolipids quantitatively in *H. pluvialis*. Lipidomics analysis will not only provide a broad context for understanding TAG biosynthesis in living cells, but will also help researchers understand membrane turnover and remodelling under high light acclimation.

RNA sequencing (RNA-seq) is a newly developed, deep-sequencing technology for analysing entire transcriptomes. High-throughput DNA sequencing can produce a library of short cDNA reads that can then be aligned to a reference genome or transcriptome, or can be assembled *de novo* without knowing the genome sequence of a given organism. This technology can provide a genome-scale transcription map that consists of the transcriptome structure and/or the level of transcriptional expression of individual genes (Wang *et al.*, 2009*b*). RNA-seq is particularly attractive for unsequenced organisms such as *H. pluvialis* because it does not rely on existing knowledge of the genome sequence for quantifying the transcriptome (Guarnieri *et al.*, 2011; Rismani-Yazdi *et al.*, 2011, 2012; Radakovits *et al.*, 2012).

In the current study, congruent lipidomic and transcriptomic analyses of *H. pluvialis* cells in two life cycle forms—macrozooids and haematocysts—were conducted to reveal its genomic and metabolic features. Special emphasis was placed on deciphering the regulation of astaxanthin biosynthesis and lipid metabolism during the encystment process under conditions of high irradiance.

## Materials and methods

### Cell growth conditions

*Haematococcus pluvialis* (UTEX #2505) cells were cultured in glass columns (4cm diameter) containing MES-Volvox (MV) medium at 25 °C under continuous illumination (20 μmol photon m^–2^ s^–1^). Cultures were aerated with sterilized air containing CO_2_ at 398 ppm and at a feed velocity of 100ml min^–1^. Initial cell density was 0.27×10^5^ cells ml^–1^. For high light level treatments, cells in the late exponential stage (2.23×10^5^ cells ml^–1^) were exposed to continuous levels of high irradiance at 400 μmol photon m^–2^ s^–1^ for 48h. Cell number was measured using a haemocytometer under light microscopy. Total chlorophyll contents were calculated using the method described by Porra (2002), and total carotenoid contents were measured using the calculation described by Lichtenthaler and Welburn (1983). For lipidomic analysis, cells were cultured under 400 μmol photon m^–2^ s^–1^ for 4 d, and cells from days 0, 1, 2, and 4 were harvested and lyophilized prior to lipid extraction and analysis.

### Measurement of ROS production from *H. pluvialis*

2’, 7’-Dichlorodihydrofluorescein diacetate (H_2_DCFDA; Sigma, St Louis, MO, USA) was used to measure ROS according to the method described by Han *et al.* (2012) with some modifications. A 5ml aliquot of 1.35×10^5^ cells ml^–1^ *H. pluvialis* was harvested by centrifugation at 1000 *g* for 5min. Cells were resuspended in 5ml of extraction buffer (50mM TRIS-HCl, pH 7.5) supplemented with 5 μM H_2_DCFDA. The suspension was incubated at room temperature in darkness for 10min. Algal cells were centrifuged at 1000 *g* for 5min at 4 °C and washed twice with 5ml of extraction buffer. To extract the intracellular DCF, 200 μl of extraction buffer was added to the cell pellet and cells were lysed by 1min of homogenization. An additional 4.8ml of extraction buffer was added and centrifuged at 2000 *g* for 8min to collect the supernatant. The supernatant liquid was diluted 1/100 and the fluorescence was measured using a fluorescence spectrophotometer LS55 (PerkinElmer, USA). The excitation and emission wavelengths were 488nm and 525nm, respectively. Fluorescence values from untreated cells were calculated to correct the fluorescence values obtained from cells treated with H_2_DCFDA. Corrected fluorescence values were then divided by cell number. Macrozooid and haematocyst cells were harvested at 0, 1, and 2 d under high light irradiance.

### Pigment measurement

For pigment extraction, 2ml cell cultures were harvested by centrifugation (10 000 *g*, 2min) and supernatants were discarded. Cells were lysed by homogenization in 0.2ml of 90% (w/w) acetone for 1min. Samples were centrifuged at 10 000 *g* for 2min to obtain the supernatants, which were then filtered through a 0.2 μm nylon filter. Filtrates were analysed on a Shimadzu Prominence high-performance liquid chromatography (HPLC) model LC-20AD equipped with a Waters Spherisorb S5 ODS2 cartridge column (4.6×250mm). HPLC analysis was conducted according to the method described by Park *et al.* (2010). Pigments were identified by retention time and absorption spectra with reference to pigment standards (DHI 14C Centralen; Denmark). Concentrations of individual pigments were determined by HPLC using chlorophyll and carotenoid standards.

### RNA preparation

*Haematococcus pluvialis* cells were frozen with liquid nitrogen and ground using a mortar and pestle. Total RNA was extracted using the Plant RNeasy Mini Kit (Qiagen, Valencia, CA, USA). Polyadenylated mRNA was isolated from 50 μg of total RNA samples using the FastTrack™ MAG Micro mRNA Isolation Kit (Life Technologies, Gaithersburg, MD, USA) according to the manufacturer’s protocol. The isolated mRNA was quantified using an Agilent RNA 6000 NANO Chip (Agilent, Wilmington, DE, USA). Subsequently, 200ng of mRNA was used for constructing the cDNA library.

### cDNA library construction

cDNA rapid library preparation began with fragmentation of mRNA using ZnCl_2_ and heat. Cleaved RNA fragments were reverse transcribed into first-strand cDNA using reverse transcriptase and random hexamer primers (Roche, Switzerland). RNA templates were removed before a replacement strand was synthesized to generate double-stranded cDNA. The double-stranded cDNA ends were polished (blunted) and short adaptors were ligated onto both ends. The adaptors provided priming sequences for both amplification and sequencing of the sample library fragments as well as the sequencing key, a short sequence of four nucleotides used by the system software for base calling. Purification with size selection was conducted using AMpure beads (Beckman Coulter, Brea, CA, USA). Lastly, the quality of the cDNA fragment library was assessed using a 2100 BioAnalyzer (Agilent) and the library was quantified to determine the optimal amount for use as template for emulsion-based clonal amplification.

### Emulsion PCR

Single ‘effective’ copies of template species from the cDNA library to be sequenced were hybridized to DNA Capture Beads. The immobilized library was then resuspended in amplification solution and the mixture was emulsified and amplified by PCR. After amplification, the DNA-carrying beads were recovered from the emulsion and enriched. The second strands of the amplification products were melted away as part of the enrichment process, leaving the amplified single-stranded DNA library bound to the beads. The sequencing primer was then annealed to the immobilized, amplified DNA templates.

### 454 FLX Titanium sequencing run

Following amplification, the DNA-carrying beads were placed in the wells of a PicoTiterPlate (PTP) device, with each well holding single-stranded DNA-containing beads. The loaded PTP was then inserted into the Genome Sequencer FLX System and exposed to sequencing reagents. Information from all wells of the PTP was captured simultaneously by a camera and was processed in real time by the onboard computer.

### Quantitative real-time PCR

Total RNA was converted to cDNA using 5X reverse transcription master pre-mix (Elpis, Korea). cDNA was amplified using SYBR pre-mix (Takara, Japan) and a Thermal Cycler Dice Real Time System TP 8200 (Takara, Japan). Reverse transcription quantitative real-time PCR (RT–qPCR) conditions were as follows: 95 °C for 10 s; 95 °C for 5 s, 55–65 °C for 30 s (40 cycles); 95 °C for 15 s, 60 °C for 30 s, 95 °C for 15 s; 72 °C for 5min; and 15 °C for 5min. To calculate relative changes in gene expression, results were analysed using the 2-ΔΔ CT method described by Livak and Schmittgen (2001).

All RT–qPCR experiments described were performed at least twice with three technical repeats (*n*=3) for independently prepared biological samples (*n*=2). Results were represented as the mean ±SD from six measurements. The actin gene was used as an internal control, as it has been used in other study (Eom *et al.*, 2006). The ratio between the expression values of each gene and the actin gene (*Act1*) in *Haematococcus* cells under normal light conditions (control) was set to 1 and the expression ratios of other conditions were given relative to the control.

### Lipid extraction from *H. pluvialis*

For lipidomics analysis, a 10mg biomass was used for lipid extraction. Total lipids were extracted by grinding the cell pellet using an ice-cold mortar and pestle in 6ml of chloroform:methanol (2:1, v/v). Lipid extracts were vigorously agitated at room temperature for 1h. Extracts were then mixed with 1.5ml of potassium chloride (0.7%, w/v). Following centrifugation at 1000 *g* for 5min, extracts were separated into two phases and total lipids in the lower organic phase were transferred into another vial using a glass pipette. The organic solvent was evaporated under a stream of nitrogen.

### Lipidomic analysis

Total lipids were recovered in 1ml of chloroform:methanol (1:1). Lipidomic analysis was performed on a 6460 triple quadruple electrospray ionization mass spectrometer (ESI/MS) coupled with a 1260 high-performance liquid chromatograph (Agilent). Single-stage mass spectrometry (MS) and MS/MS analyses in the positive or negative ion mode were conducted to identify lipid molecular species. Based on the fragmentation of lipid molecules, multiple reaction monitoring was developed and used for quantification. A 10 μl aliquot of lipid extract was mixed with internal standards and chloroform:methanol (1:1) to a final volume of 50 μl. For positive ion mode analysis, the internal standards mixture provided 2 μM digalactosyldiacylglycerol (DGDG; 18:0/18:0), 5 μM monogalactosyldiacylglycerol (MGDG; 18:0/18:0), 0.25 μM phosphatidylcholine (PC; 18:1/18:1), 0.32 μM phosphatidylethanolamine (PE; 17:0/14:0), and 1 μM TAG (17:0/17:0/17:0). For negative ion mode analysis, the mixture contained internal standards providing 1.243 μM phosphatidylglycerol (PG; 17:0/20:4) and 1.15 μM phosphatidylinositol (PI; 17:0/20:4). Prior to infusion into the ESI/MS, 1 μl of lipid mixture was separated at 40 °C on a ZOBAX SBC18 column (1.8 μm, 2.1×150mm, Agilent) and an Extend C18 column (1.8 μm, 2.1×150mm, Agilent) for positive and negative mode MS analyses, respectively. For positive mode, the mobile phases were methanol:acetonitrile:H_2_O (19:19:2; A) and isoproponal (B) containing 0.1% formic acid and 10mM ammonium acetate. The liquid chromatography (LC) gradients were as follows: 0min, 90% A and 10% B; 5min, 90% A and 10% B; 25min, 60% A and 40% B; 60min, 45% A and 55% B; 66min, 45% A and 55% B; and 68min, 90% A and 10% B. For negative mode, the mobile phases were 85% methanol (A) and isopropanol (B) containing 0.025% NH_4_OH. The LC gradients were as follows: 0min, 95% A and 5% B; 15min, 85% A and 15% B; 22min, 45% A and 55% B; 42min, 45% A and 55% B; and 44min, 95% A and 5% B. The flow rate was 0.2ml min^–1^. Lipid standards for the membrane glycerolipids were purchased from Avanti Polar Lipids (Alabaster, AL, USA) and TAG standards were purchased from Sigma-Aldrich. For lipidomics analysis, three biological replicates (*n*=3) were included. For each biological replicate, lipids were extracted in duplicate for independent LC/MS analysis. Lipid data were presented as the mean ±SD from six measurements for each time point.

## Results and Discussion

### Morphological and biochemical changes during encystment under high irradiance

**Fig. 1.**
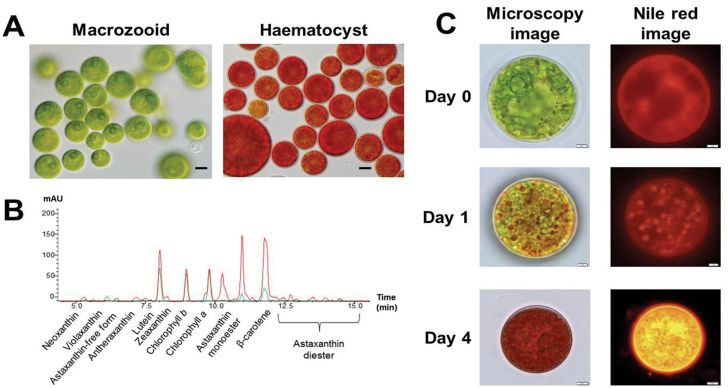
*Haematococcus pluvialis* cell. (A) Light microscopy image of *H. pluvialis*. Left panel, macrozooid cells (control); right panel, haematocyst cells (high light stressed for 2 d). Scale bar=10 μm. (B) HPLC pigment analysis of *H. pluvialis* cells. Green and red lines indicate the macrozooid cells and high light-stressed haematocysts, respectively. (C) Nile red-stained images of *H. pluvialis* cells before and after transferring them to high light stress conditions. Scale bar=2 μm.

The life cycle of *H. pluvialis* consists of four distinct types of cells: microzooids, motile macrozooids, non-motile palmelloids, and haematocysts (Elliot, 1934). Under favourable conditions, *H. pluvialis* cultures were dominated by macrozooids (Fig. 1A); under conditions of high light intensity (400 μmol photon m^–2^ s^–1^), 2 d cultures of *H*. *pluvialis* were transformed into haematocysts while accumulating large amounts of carotenoids (Fig. 1B; Supplementary Table S2 available at *JXB* online). The total carotenoid content increased from 1.5 μg ml^–1^ to 6.22 μg ml^–1^ after 2 d of exposure to high irradiance conditions, while total chlorophyll contents were reduced from 2.32 μg ml^–1^ to 1.60 μg ml^–1^ (Supplementary Table S2 available at *JXB* online). In green macroozoid cells, chlorophyll *a* and *b* and lutein were the most abundant pigments, followed by β-carotene. In contrast, astaxanthin acyl-esters and β-carotene were the prevailing pigments in haematocysts, followed by lutein and chlorophyll *a* and *b*. As indicated by fluorescence emitted after Nile red staining, cells exposed to high light formed a large number of carotenoid-rich lipid bodies in the cytosol, a striking oleaginous characteristic of haematocysts (Fig. 1C).

**Fig. 2.**
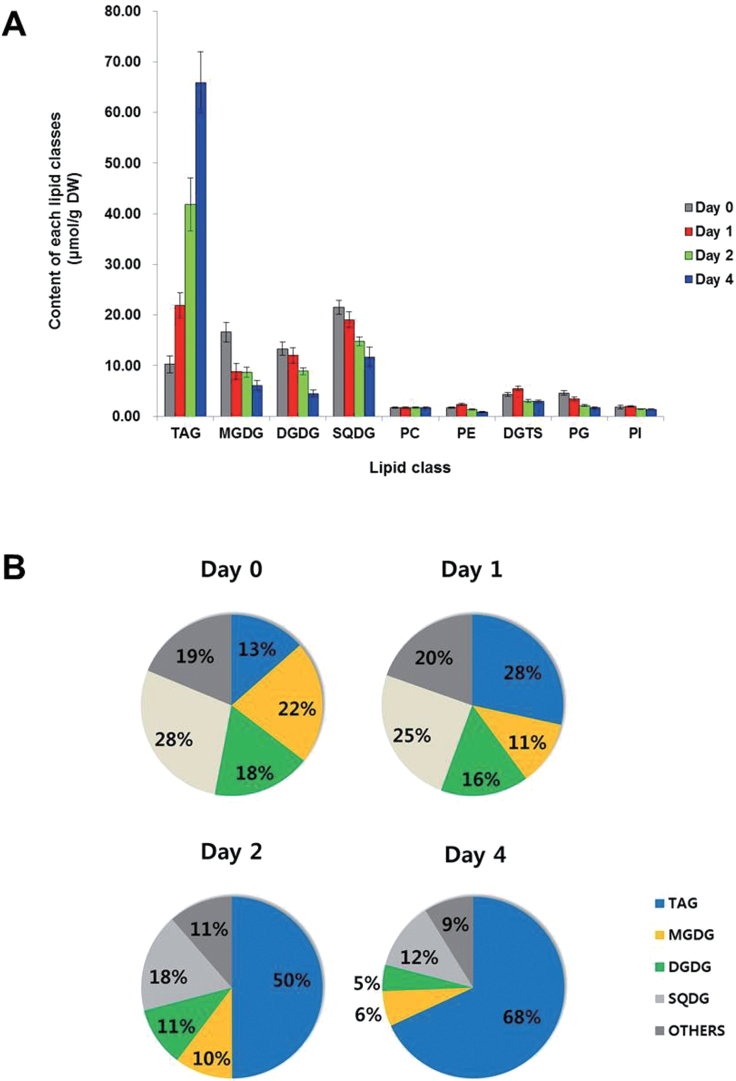
Changes in total content of the six major lipid classes under high light irradiation of *H. pluvialis*. (A) Content of each glycerolipid class of *H. pluvialis* under high light irradiation. (B) Lipid composition changes in *H. pluvialis* under high light irradiance. Values represent the mean ±SD (*n*=3). TAG, triacylglycerol; MGDG, monogalactosyldiacylglycerol; DGDG, digalactosyldiacylglycerol; SQDG, sulphoquinovosyl diacylglycerol; PC, phosphatidylcholine; PE, phosphatidylethanolamine; DGTS, diacylglyceryltrimethylhomoserine; PG, phosphatidylglycerol; PI, phosphatidylinositol.

To investigate cellular alterations in glycerolipid composition and content during encystment, glycerolipidomes of *Haematococcus* cells exposed to high light were analysed quantitatively. The total content of glycerolipids increased from 75.99 μmol g^–1^ DW to 96.80 μmol g^–1^ DW following exposure of *H. pluvialis* cells to high light for 4 d (Fig. 2A). Glycolipids including MGDG, DGDG, and sulphoquinovosyl diacylglycerol (SQDG), the main constituents of the chloroplast membranes and photosynthetic complexes, were the major lipid classes in macrozooid cells (day 0) and accounted for up to 67.8% of total glycerolipids. Among the extraplastidic membrane glycerolipids including diacylglyceryltrimethylhomoserine (DGTS), PC, PI, and PE, DGTS was the most abundant (~5.6% of total glycerolipids). The presence of two structural analogues, DGTS and PC, distinguished *H. pluvialis* from *Chlamydomonas reinhardtii*, which does not contain PC in its lipidome (Yoon *et al.*, 2012). After 4 d under high light, TAG in macrozooid cells increased from 10.26 μmol g^–1^ DW (13.5% of total glycerolipids) to 65.91 μmol g^–1^ DW (67.8% of total glycerolipids). A concomitant and remarkable decrease of MGDG, DGDG, and SQDG and slight reductions of PE, DGTS, and PG were also observed, while PC and PI remained stable (Fig. 2B). For example, MGDG and DGDG accounted for 21.9% (corresponding to 16.64 μmol g^–1^ DW) and 17.6% (13.35 μmol g^–1^ DW) of total glycerolipids in green macrozooid cells, and decreased to 6.3% (6.11 μmol g^–1^ DW) and 4.7% (4.52 μmol g^–1^ DW) of total glycerolipids, respectively, in haematocyst cells after 4 d of high light induction. SQDG decreased from 21.53 μmol g^–1^ DW (28.3% of total glycerolipids) to 11.73 μmol g^–1^ DW (12.1% of total glycerolipids) after 4 d under high irradiation (Fig. 2A).

**Fig. 3.**
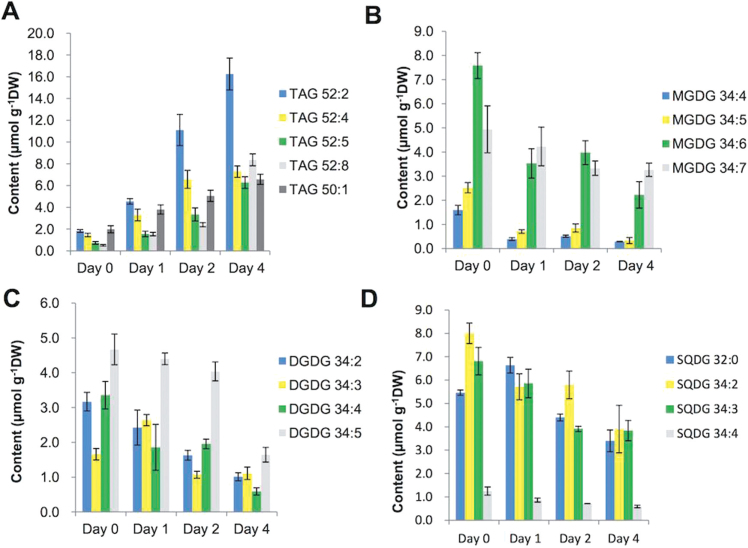
Changes in composition of TAG (A), MGDG (B), DGDG (C), and SQDG (D) in *H. pluvialis* under high light irradiation. Acyl chains of glycerolipid molecular species are described by the convention carbon number:number of double bonds. Values represent the mean ±SD (*n*=3). TAG, triacylglycerol, MGDG, monogalactosyldiacylglycerol, DGDG, digalactosyldiacylglycerol, SQDG, sulphoquinovosyl diacylglycerol.

As shown in Fig. 3, high irradiance induction exerted a profound impact on the cellular content of individual TAG species and molecular species of glycolipids. The major TAG species (52:2, 52:4, 52:5, 52:8, and 50:1) accounted for >50% of total TAG, among which TAG 52:2 was the most predominant, accounting for 24.7% of total TAG (Fig. 3A). Eight species (50:2, 50:3, 50:9, 52:1, 52:6, 52:7, 52:9, and 54:10) were designated as the minor TAG species, which accounted collectively for 20–40% of total TAG. The other 11 TAG species, accounting for <5% of total TAG, were defined as trace TAG molecular species (Supplementary Fig. S1 available at *JXB* online). All TAG species increased in response to high irradiance conditions.

Four species of MGDG were detected, namely 34:4, 34:5, 34:6, and 34:7. MGDG 34:4, 34:5, and 34:6 decreased dramatically after 1 d under high irradiance and then decreased more moderately from day 2 to day 4, whereas 34:7 remained at a steady level over 4 d (Fig. 3B). In contrast to MGDG, the molecular species of the bilayer glycerolipid DGDG exhibited complex responses to high irradiance induction. For example, among four DGDG species, DGDG 34:2 and 34:4 progressively decreased over 4 d under high irradiance conditions, 34:3 slightly increased on day 1 and then declined to a level lower than the basal level (day 0), and DGDG 34:5 was not affected during the first 2 d and then decreased by 3-fold in the following 2 d (Fig. 3C). Three major SQDG species (32:0, 34:2, and 34:3) decreased substantially when induced by high irradiance conditions, while the minor species SQDG 34:4 decreased only slightly (Fig. 3D). The profiles of PG, PC, DGTS, PI, and PE from *Haematococcus* cells exposed to high irradiation conditions are presented in Supplementary Fig. S2 available at *JXB* online.

### Sequencing and *de novo* assembly of the *H. pluvialis* transcriptome

The cDNA libraries for macrozooids and haematocysts were independently prepared twice to minimize any biological variations. Four pyrosequencing runs using a Roche GS-FLX 454 yielded a total of 2 141 048 raw reads, equivalent to ~848Mb of data for the *H. pluvialis* transcriptomes. The average length of the raw sequencing reads was ~396bp. The pre-assembly process of trimming and cleaning for quality control yielded a total of 2 139 711 high quality reads, which were further assembled into 1 453 995 contiguous sequences (contigs). A total of 17 364 reads remained as singletons (reads not incorporated into the assembly). The average length of contigs was 589bp, and overlapping contig reads were further assembled into 34 947 isotigs. The average length of the isotigs was 1555 bases with an N50 of 1737 bases, meaning that 50% of the assembled bases were incorporated into isotigs >1737 bases. A status summary of the sequencing and assembly results is presented in Table 1 and the size distributions for raw reads, assembled contigs, isotigs, and sequence coverage are presented in Supplementary Fig. S3 available at *JXB* online. Isotigs sharing common contigs were clustered into individual isogroups, with each presumably representing one gene locus capable of producing multiple, alternatively spliced transcripts. Lastly, the *de novo* transcriptome assembly identified a total of 18 482 isogroups, possibly representing the total number of genes in the *H*. *pluvialis* transcriptome under the given culture conditions. The 6937 isogroups contained multiple isotigs (equivalent to an average of 1.89 transcripts per gene), while 11 545 isogroups had only one isotig or contig each. Raw data files are available at the National Center for Biotechnology Information (NCBI) Sequence Read Archive (accession no. SRR1040551).

**Table 1.** *Summary of* de novo *assembly in* H. pluvialis

Sequencing	No. of sequences	Bases
Raw sequencing reads	2 141 048	848 713 419
Average read length		396
Pre-assembly		
Trashed	1337	3 795 275
Reads used in assembly	2 139 711	844 918 144
Average read length		395
Assembly		
Contigs		
Reads assembled as contigs	1 453 995	558 385 452
Number of contigs	62 636	36 715 613
Average length of contigs		589
Isotigs		
Number of isotigs	34 947	54 330 087
Average length of isotigs		1555
Isotig N50		1737
Number of isogroups	18 482	
Singletons	17 364	

### Functional genome annotation

**Fig. 4.**
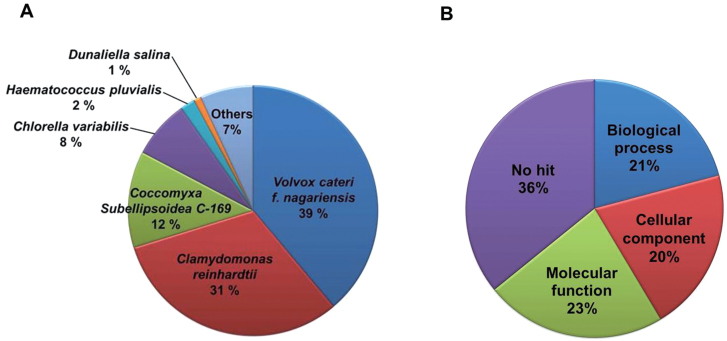
Top-hit species distribution of BLAST matches and gene ontology (GO) analysis of *H. pluvialis*. (A) Total BLAST matches were clustered to related green microalgae species, *Volvox carteri* f. *nagariensis* (39%) and *Chlamydomonas reinhardtii* (31%). (B) Isotigs and contigs of *H. pluvialis* were categorized in three classes: molecular function (23%), biological process (21%), and cellular component (20%). (This figure is available in colour at *JXB* online.)

All unique sequences (total of 267 contigs and 34 680 isotigs) were queried against the sequences available in the NCBI non-redundant protein database using the BLASTx algorithm for annotation. Approximately 57.9% of the BLAST queries (20 234 unique sequences) produced significant blast matches with expected values (E-values) below a threshold of 10^–6^. The top-hit species distribution of BLAST matches revealed distinct characteristics of the microalgal transcripts. For example, 70% of the total BLAST matches were clustered to related microalgal species, predominantly *Volvox carteri* f. *nagariensis* (39%) and *C. reinhardtii* (31%) (Fig. 4A).

The best hits (sequences producing the highest scores/lowest E-values) selected from the BLAST queries were further annotated with gene ontology (GO) terms through the GO site (http://www.geneontology.org/). The GO database represents ontological descriptions for gene products according to three main categories: biological processes, cellular components, and molecular functions. A total of 61 GO terms were assigned to 22 431 cDNAs, including isotigs and contigs. Among all GO terms extracted, 7959 (23%) belonged to the molecular functions class and 7291 (21%) and 7181 (20%) were grouped into the biological processes and cellular components classes, respectively (Fig. 4B).

Of the assembled isotigs and contigs, 36% were not subject to standardized GO classification because they did not match any sequence in the GO database. Such a large quantity of ‘no hit’ sequences may have been attributable to a lack of transcriptome data or gene prediction data of similar species in the GO database. Of the remaining assembled isotigs and contigs, a significant number of sequences were associated with biological processes referred to as metabolic process (26% of GO-annotated sequences searched), followed by those that were unclassified (16%), those referred to as cellular process (12%), and those associated with biological regulation (11%). As for those sequences associated with molecular functioning, genes related to binding (39%) and catalytic activity (35%) were the most abundant, followed by those that were unclassified (12%). In the cellular components category, ~39% of the transcripts belonged to cell part, 12% were unclassified, 12% belonged to organelle, and 9% to membrane. A detailed analysis of GO terms of the three function classes is presented in Supplementary Fig. S4 available at *JXB* online.

**Fig. 5.**
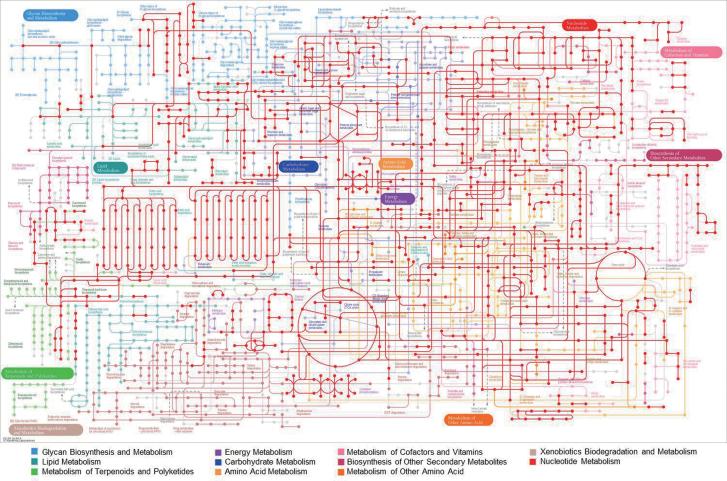
Kyoto Encyclopedia of Genes and Genomes (KEGG) pathway analysis of *H. pluvialis* transcripts. Isotigs and contigs of expected values (E-values) below the threshold of 10^–6^ were analysed. *Haematococcus pluvialis* expressed transcripts for enzymes functioning in most of the major metabolic pathways, including *N*- or *O*-glycan, lipid, carbohydrate, energy, amino acid, nucleotide, cofactor and vitamin, and isoprenoid metabolism.

Kyoto Encyclopedia of Genes and Genomes (KEGG) pathway analysis is an alternative approach to categorizing gene functions with an emphasis on biochemical pathways. To reconstruct the metabolic pathways in *H*. *pluvialis*, all assembled unique sequences were queried against the KEGG database and 22.4% of queries were assigned to KEGG orthology (KO) identifiers (ID) with E-values below the threshold value of 10^–6^. These KO IDs were mapped against all pathway maps through the basic pathway mapping tool of the KEGG mapper (http://www.genome.jp/kegg/mapper.html). As shown in Fig. 5, *H*. *pluvialis* expressed transcripts for enzymes functioning in most of the major metabolic pathways, including metabolism of *N*- or *O*-glycans, lipids, carbohydrates, energy, amino acids, nucleotides, cofactors and vitamins, and isoprenoids. However, transcriptional expression of most of the genes related to certain pathways—such as the biosynthetic pathways for glycosphingolipids, phenylpropanoids, and alkaloids, and xenobiotic biodegradation and metabolism—were not detected.

### Comparison of global transcriptomes of macrozooids and haematocysts

**Fig. 6.**
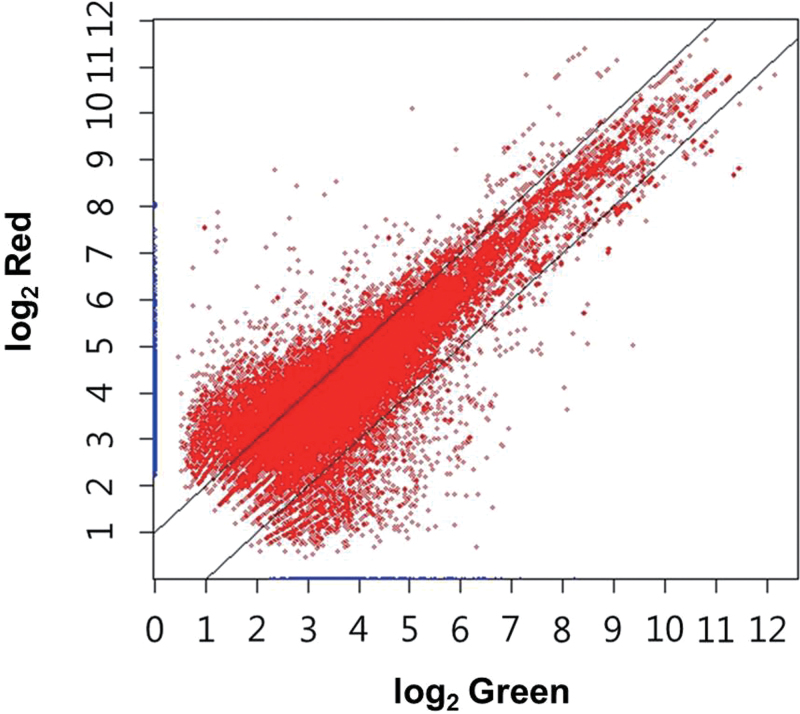
Scatter plot of normalized abundances of each cell type [green (macrozooid) and red (haematocyst)]. Differential expression >2-fold was detected in as many as 38.1% of assemblages between macrozooid and haematocyst cells. Approximately 30.6% of the transcripts were expressed more abundantly in haematocyst cells than in macrozooid cells. (This figure is available in colour at *JXB* online.)

Normalized abundances of individual transcripts (assembled sequences including isotigs and contigs) in green macrozooids and haematocysts are presented in a scatter plot in Fig. 6. Over 2-fold differential expression was detected in as many as 38.1% of the assembled sequences (13 313 out of 34 947) between the two cell types. Surprisingly, ~30.6% of the transcripts (10 705/34 947) were expressed more abundantly in haematocysts than in macrozooids. In contrast, only 7.5% (2608/34 947) of the transcripts were expressed in higher amounts in macrozooids than in haematocysts. As two independently prepared cDNA library sets were available, the same analysis was performed separately for each set, confirming that haematocysts up-regulated many more genes than green macrozooid cells. Multiple lines of evidence indicated specificity regarding the high irradiance-induced up-regulation of many genes of great diversity (as much as ~30% of all unique sequences analysed). First, the alteration pattern of many sets of genes revealed good agreement with the known physiology of *Haematococcus* cells under high light conditions. For example, the coordinated and apparent increase in transcript abundance was noticeable in many genes related to protection against photooxidative stress. Conversely, the expression of a number of highly active genes in macrozooids, especially genes encoding proteins involved in light-harvesting activity, declined under high irradiance conditions. Secondly, the extent of up-regulation varied among different pathways/genes. For example, the *de novo* fatty acid biosynthesis pathway was up-regulated to a greater extent than the TAG assembly pathway under high irradiance conditions. Thirdly, the transcriptome data suggested that >61% of genes were not responsive to high irradiance, such as those involved in the cell cycle, carbon fixation, and chlorophyll metabolism, etc. Lastly, most of the multiple representative housekeeping genes in the next-generation sequencing (NGS) remained steady in their expression under high irradiance (Supplementary Table S3 available at *JXB* online). In fact, the expression of >30 different kinds of genes which have been used as housekeeping gene controls in various studies was examined between macrozooids and haematocysts in the NGS data, and the slope value (0.9372) of the correlation graph demonstrated that the expression levels of each gene between the two kinds of cells was quite similar overall (Supplementary Fig. S5 available at *JXB* online).

The assembled sequences that showed differential expression (>2-fold) between haematocysts and macrozooids were mapped on the KEGG reference pathway diagram (Supplementary Fig. S6 available at *JXB* online). The KEGG analysis revealed that those sequences up-regulated in haematocysts included various genes encoding proteins involved in a wide range of metabolic processes in addition to the genes for the synthesis of astaxanthin and oil, the two major products of haematocysts. For example, many genes coding for various metabolic enzymes, particulary for the biosynthesis of secondary metabolites, microbial metabolism in diverse environments, and nucleic acid and amino acid metabolism were more highly expressed in *Haematococcus* cells under high irradiance conditions. Additionally, genes up-regulated in haematocysts were enriched with those known to be involved in processes related to gene expression, such as spliceosome formation, RNA transport, ribosome biogenesis, and protein processing. Such an increase in abundance of those transcripts related to the information flow may aid the active translation of the transcripts of the diversity under high light conditions. The present NGS data analysis of haematocyst transcripts also indicated that most of the genes involved in basic metabolism such as glycolysis, the tricarboxylic acid (TCA) cycle, and oxidative phosphorylation were expressed at levels comparable with those of macrozooids and that few genes in those pathways were even up-regulated. Collectively, this comparative transcriptomic analysis suggested that haematocysts, previously referred to as resting cells, were probably not quiescent at all, but instead were probably more metabolically active than macrozooids.

### Analysis of the transcriptome involved in astaxanthin biosynthesis

Multiple lines of transcriptome evidence indicated that synthesis of the five-carbon isoprene units in *H*. *pluvialis* was most probably mediated by the MEP pathway rather than the MVA pathway (Supplementary Fig. S7 available at *JXB* online). For example, transcripts for all eight genes involved in the MEP pathway were identified in *Haematococcus* cells. Four of the MEP pathway genes exhibited higher transcript levels in haematocysts—as much as ~1.8- to 2-fold of that in macrozooids—which correlated with the accumulation of astaxanthin. The four up-regulated genes were those encoding 1-deoxy-d-xylulose-5-phosphate synthase (DXS), 1-deoxy-d-xylulose 5-phosphate reductoisomerase (DXR), 1-hydroxy-2-methyl-2-(E)-butenyl-4-diphosphate synthase (HDS), and 4-hydroxy-3-methylbut-2-enyl diphosphate reductase (HDR), which showed the highest fold change. In contrast, only two types of MVA pathway genes, namely one encoding acetyl-CoA C-acetyltransferase (ACAT) and two encoding hydroxymethylglutaryl-CoA (HMGS), were identified in the transcriptome of either haematocysts or macrozooids. Of these putative MVA pathway genes, only one of the *HMGS* genes was up-regulated at the transcript level, whereas the others did not change under high irradiance conditions.

**Fig. 7.**
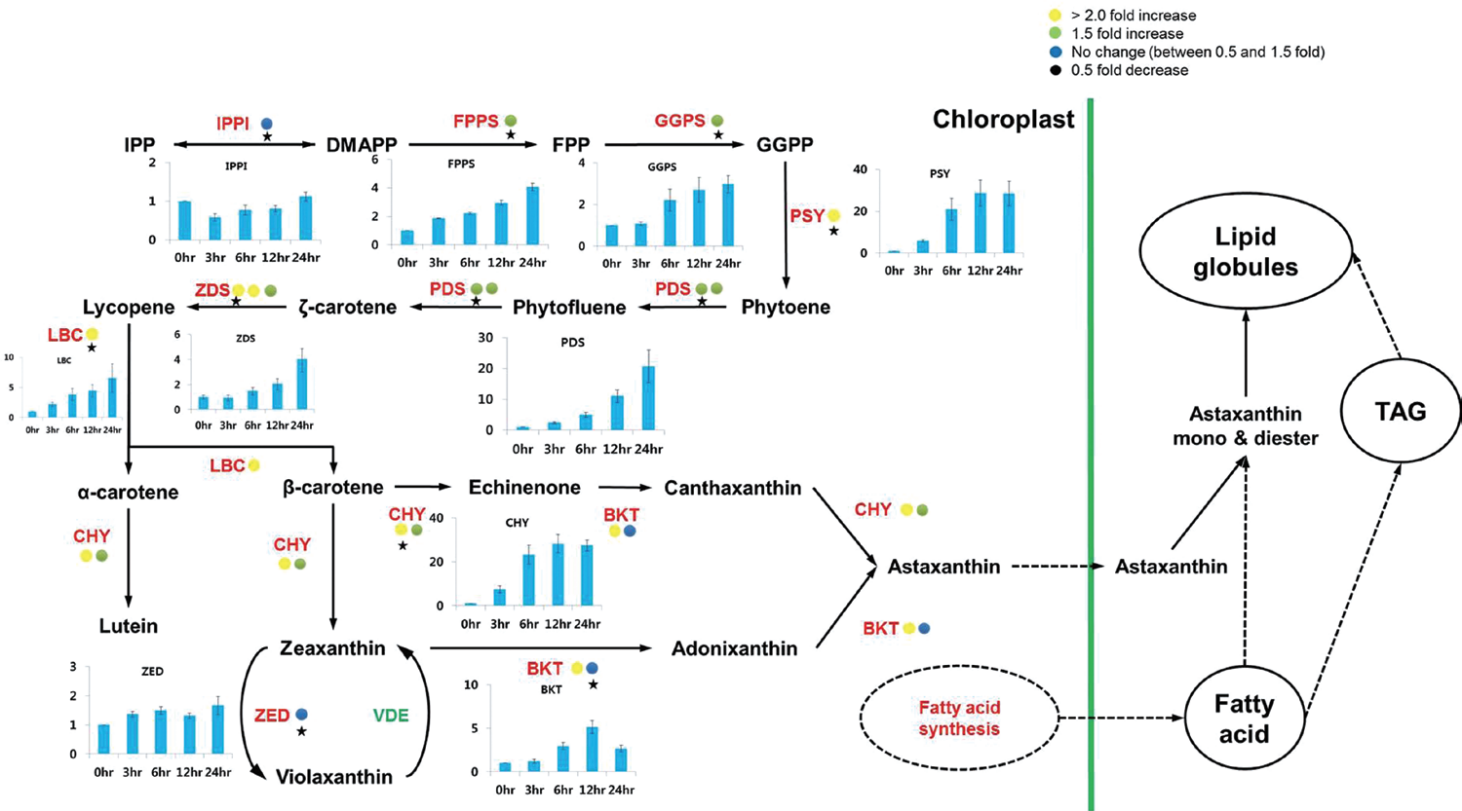
Differential expression of genes involved in the astaxanthin biosynthesis pathway. Coloured circles indicate the normalized read number ratio of red (haematocyst) per green (macrozooid) cells and each circle represents an independent isogroup (gene). The star designates the primer-designed gene for RT–qPCR. The bar graph indicates the expression pattern of each star-marked gene in the RT–qPCR analysis. Values in the bar graphs are the mean ±SD of six different measurements. IPPI, isopentenyl diphosphate isomerase; FPPS, farnesyl diphosphate synthase; GGPS, geranylgeranyl diphosphate synthase; PSY, phytoene synthase; PDS, phytoene desaturase; ZDS, zeta-carotene desaturase; LBC, lycopene beta-cyclase; CHY, carotenoid hydroxylase; BKT, beta-carotene ketolase.

DMAPP synthesis is thought to be catalysed by isopentenyl diphosphate isomerase (IPPI) (Sun *et al.*, 1998). However, the transcriptomic analysis indicated that neither of the two putative IPPI transcripts was up-regulated during the cellular accumulation of astaxanthin (Fig. 7). The results of previous studies have suggested that HDR was an additional enzyme capable of catalysing interconversion between IPP and DMAPP (Hoeffler *et al.*, 2002; Rohdich *et al.*, 2002). One of the two HDR-encoding transcripts was moderately up-regulated (>2-fold) under high irradiance (Supplementary Fig. S7 available at *JXB* online), indicating that this enzyme was more likely to be responsible for supplying the isoprene units for astaxanthin synthesis under high light conditions in this organism.

The astaxanthin biosynthetic pathway from IPP and DMAPP is presented in Fig. 7. All of the genes essential for astaxanthin synthesis from DMAPP were up-regulated in concert under high irradiance with the exception of *IPPI*. As the first committed step in the pathway, phytoene biosynthesis has long been considered a bottleneck in carotenoid biosynthesis (Lorenz and Cysewski, 2000; Cardozo *et al.*, 2007). Previously, the expression of the phytoene synthase (*PSY*) gene in *Haematococcus* cells was shown to be up-regulated by high light levels (Steinbrenner and Linden, 2003; Vidhyavathi *et al.*, 2008). Of the four phytoene synthase cDNA sequences, including partial sequences reported for *H*. *pluvialis* in GenBank, the present analysis confirmed that only one was up-regulated in haematocysts. The gene encoding phytoene desaturase (PDS) that catalyses the first step in lycopene biosynthesis was also up-regulated at the mRNA level under high irradiance (Steinbrenner and Linden, 2003; Steinbrenner and Sandmann, 2006). The transcriptome analysis demonstrated that two *PDS* genes were up-regulated by ~1.5- to 1.9-fold in haematocysts compared with the macrozooids. The plastid terminal oxidase (PTOX) has been suggested to serve as a cofactor for desaturation of phytoene and β-carotene (Li *et al.*, 2008). Previously, two *PTOX* genes (*PTOX1* and *2*) have been reported in *H*. *pluvialis* (Wang *et al.*, 2009*a*; Houille-Vernes *et al.*, 2011), and our RNA-seq analysis identified their transcripts. As shown in Table 2, transcripts of the *PTOX1* and *2* genes increased >2-fold under high light level conditions. Consistent with previous observations (Steinbrenner and Linden, 2003), the transcripts of the *Haematococcus* lycopene β-cyclase gene were >2-fold more abundant in haematocysts than in macrozooids. It is worth noting that expression of the ε-cyclase and zeaxanthin epoxidase genes was either undetectable or slightly reduced, suggesting that lycopene is largely shunted into β-carotene synthesis as part of astaxanthin synthesis. Subsequent oxygenation reactions converting β-carotene to astaxanthin are considered rate-limiting steps catalysed by β-carotene ketolase (BKT) and β-carotene hydroxylase (CHY) (Steinbrenner and Linden, 2001, 2003; Vidhyavathi *et al.*, 2008). The present NGS data showed that these two genes were also up-regulated in haematocysts.

**Table 2.** Isotigs of ROS scavenging-related genes in the *H. pluvialis* transcriptome

Isogroup	Isotig number	Description	Accession	Read ratio (red/green)
isogroup04416	isotig18119–18120	Catalase (*Haematococcus pluvialis*)	ABK41476.1	1.40
isogroup00630	isotig07317–07322	Mn superoxide dismutase 4 (*Chlamydomonas reinhardtii*)	ACZ37254.1	1.07
isogroup05851	isotig20989–20990	Mn superoxide dismutase (*Haematococcus pluvialis*)	AAW69292.1	1.08
isogroup05972	isotig21231–21232	Mitochondrial Mn superoxide dismutase (*Haematococcus pluvialis*)	ABF82431.1	1.67
isogroup11662	isotig28274	Chloroplast Fe superoxide dismutase 1 precursor (*Chlamydomonas reinhardtii*)	XP_001690591.1	1.15
isogroup04801	isotig18889–18890	l-Ascorbate peroxidase (*Volvox carteri f. nagariensis*)	XP_002957861.1	1.78
isogroup11649	isotig28261	l-Ascorbate peroxidase (*Chlamydomonas reinhardtii*)	XP_001695476.1	2.37
isogroup00937	isotig08842–08845	Glutathione reductase (*Coccomyxa subellipsoidea* C-169)	EIE18555.1	1.48
isogroup04339	isotig17965–17966	Glutathione reductase (*Chlamydomonas reinhardtii*)	XP_001696579.1	1.58
isogroup05030	isotig19347–19348	Chloroplast glutathione reductase (*Solanum lycopersicum*)	NP_001234243.1	3.82
isogroup04010	isotig17307–17308	Plastid terminal oxidase (*Haematococcus pluvialis*)	ABV72392.1	2.11
isogroup04343	isotig17973–17974	Plastid terminal oxidase (*Haematococcus pluvialis*)	ABV72391.1	2.07
isogroup08668	isotig25280	Monodehydroascorbate reductase (*Coccomyxa subellipsoidea* C-169)	EIE20462.1	0.79
isogroup21067	isotig37679	Dehydroascorbate reductase (*Chlamydomonas reinhardtii*)	XP_001698375.1	10.84

Collectively, almost all of the genes involved in astaxanthin biosynthesis from IPP were found to be simultaneously up-regulated in transcriptional expression under high irradiance (Fig. 7). The concerted reactions of the multiple enzymatic steps in carotenogenesis have already been demonstrated as critical for the enhancement of astaxanthin biosynthesis. For instance, the introduction of yeast astaxanthin biosynthesis genes in *Saccharomyces cerevisiae* successfully enhanced the accumulation of astaxanthin and its intermediates in transgenic cells (Ukibe *et al.*, 2009). Up-regulation of the carotenogenic genes responsible for astaxanthin biosynthesis from GGPP also led to excessive astaxanthin accumulation in a mutant strain of *Phaffia rhodozyma* (Miao *et al.*, 2011). Therefore, the greater transcript abundance of the multiple genes involved in astaxanthin biosynthesis may contribute collectively to the observed increase in astaxanthin synthesis under high irradiance. In fact, the additional RT–qPCR analysis revealed that up-regulation of all genes involved in astaxanthin biosynthesis occurred during the first 24h under high irradiance. The most dramatic increases in transcript abundance (up to 20- to 30-fold) were observed in genes such as *PSY*, *PDS*, and *CHY*. The high level of responsiveness of these genes was considered indicative of their prominent regulatory roles in augmenting astaxanthin biosynthesis.

### Transcriptome analysis related to lipid metabolism in *H. pluvialis*

Under stress conditions, many green microalgae can increase the number and size of their lipid bodies, which are discrete TAG- and carotenoid-containing organelles surrounded by a lipid monolayer (Thompson, 1996; Boussiba, 2000; Grunewald *et al.*, 2001). During cyst formation under stress conditions, *H*. *pluvialis* accumulates large amounts of TAG in extraplastidic lipid bodies, accompanied by astaxanthin deposition. Results from previous studies of the unicellular microalga *Dunaliella bardawil* demonstrated that β-carotene accumulation was closely correlated with TAG synthesis and oil globule formation (Katz *et al.*, 1995; Rabbani *et al.*, 1998). Such sink activity of oil globules for secondary carotenoids is assumed to be critical for the massive accumulation of pigments in algal cells, as it may prevent end-product inhibition of the carotenoid biosynthetic pathway (Rabbani *et al.*, 1998). Zhekisheva *et al.* (2002) previously found that *H*. *pluvialis* actively synthesized fatty acids *de novo*—mainly oleic, palmitic, and linoleic acids—and deposited them primarily in the form of TAGs and astaxanthin esters. Accumulation of astaxanthin in these algal cells was also positively correlated with that of newly synthesized oleic acid (Zhekisheva *et al.*, 2002).

**Fig. 8.**
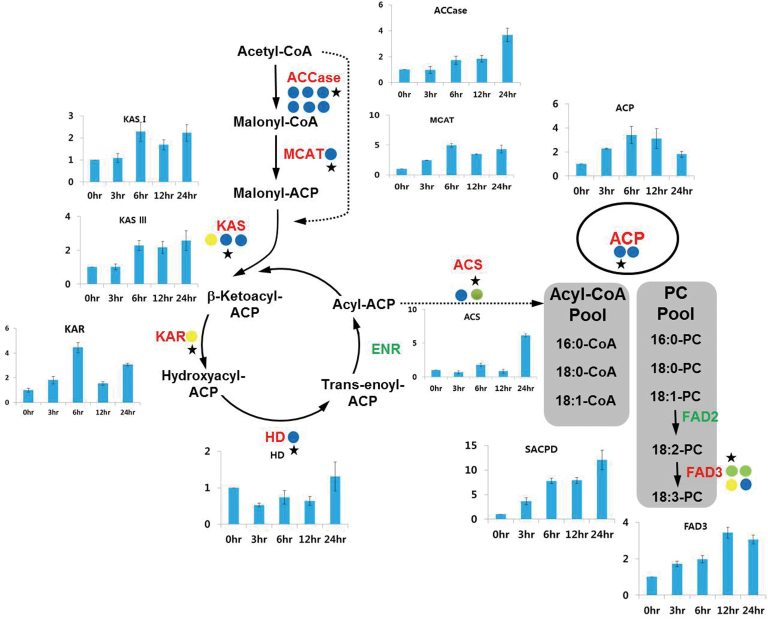
Differential expression of genes involved in the fatty acid biosynthesis pathway. Coloured circles indicate the normalized read number ratio of red (haematocyst) per green (macrozooid) cells and each circle represents an independent isogroup (gene). The star designates the primer-designed gene for RT–qPCR. The bar graph indicates the expression pattern of each star-marked gene in RT–qPCR analysis. Values in the bar graphs are the mean ±SD of six different measurements. ACC, acetyl-CoA carboxylase; MCAT, malonyl-CoA:ACP transacylase; KAS, 3-ketoacyl-ACP-synthase; KAR, 3-ketoacyl-ACP-reductase; HD, 3-hydroxyacyl-ACP dehydratase; ENR, enoyl-AC reductase; ACS, acetyl-CoA synthetase; ACP, acyl carrier protein; FAD, fatty acid desaturase.

Figure 8 shows the microalgal fatty acid synthetic process in the chloroplast. In the first committed step of fatty acid synthesis, acetyl-CoA is converted to malonyl-CoA precursors by acetyl-CoA carboxylase (ACCase). This is followed by a series of successive condensation reactions, ultimately resulting in the production of an acyl-acyl carrier protein (ACP). The transfer of a malonyl group from malonyl-CoA to the ACP is catalysed by malonyl-CoA:ACP transacylase (MCAT), forming malonyl-ACP. An acyl-ACP chain is successively elongated by a subsequent series of condensation reactions catalysed by 3-ketoacyl-ACP-synthase (KAS), 3-ketoacyl-ACP-reductase (KAR), 3-hydroxyacyl-ACP dehydratase (HD), and enoyl-AC reductase (ENR), yielding C16:0- and C18:0-ACP. In the *de novo* fatty acid biosynthesis process, a double bond can be introduced to the Δ9 position of C18:0-ACP by a stearoyl-ACP desaturase (SACPD) to form C18:1-ACP. Termination of elongation can be mediated either by an acyl-ACP thioesterase (FAT)—resulting in free fatty acid release and export to the cytosol—or by direct transfer of the acyl group to glycerol-3-phosphate and/or monacylglycerol-3-phosphate in the TAG biosynthetic pathway (Ohlrogge and Browse, 1995; Hu *et al.*, 2008). In the transcriptome analysis related to fatty acid biosynthesis, all transcripts with the exception of ENR were identified, including single genes for MCAT, KAS I, KAS III, KAR, HD, SACPD, and FAT, two genes for KAS II and ACP, and six genes for ACCase. Among those genes, the levels of KAR and KAS I transcripts were increased to the greatest degree (2.75- and 3.5-fold, respectively) in response to conditions of high light. Expression levels of ACCase, MCAT, KAS II, III, HD, and SACPD were similar between haematocysts and macrozooids. However, RT–qPCR analysis revealed that the fatty acid biosynthetic genes, with the exception of HD, were up-regulated at least 2- to 15-fold in their expression during 24h of high irradiation conditions (Fig. 8). Collectively, the results of the gene expression analyses suggested that high irradiance stimulated fatty acid biosynthesis in either a transient or progressive manner in *H. pluvialis*.

**Fig. 9.**
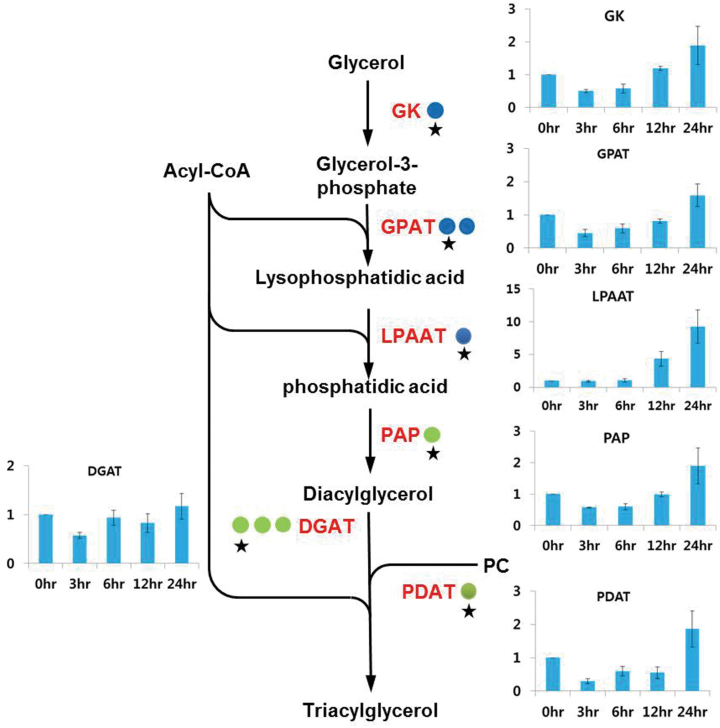
Differential expression of genes involved in the triacylglycerol biosynthesis pathway. Coloured circles indicate the normalized read number ratio of red (haematocyst) per green (macrozooid) cells and each circle represents an independent isogroup (gene). The star designates the primer-designed gene for RT–qPCR. The bar graph indicates the expression pattern of each star-marked gene in RT–qPCR analysis. Values in the bar graphs are the mean ±SD of six different measurements. GK, glycerol kinase; GPAT, glycerol-3-phosphate acyltransferase; LPAAT, lysophosphatidic acid acyltransferase; PAP, phosphatidic acid phosphatase; DGAT, diacylglycerol acyltransferase; PDAT, phospholipid:diacylglycerol transferase.

Multiple pathways for TAG biosynthesis have been elucidated in eukaryotes. The acyl-CoA-dependent pathway involves three sequential transfers of a fatty acyl moiety from acyl-CoA, the last of which is acylation of a diacylglycerol (DAG) with an acyl-CoA to form TAG. The first fatty acid transfer to position 1 of glycerol-3-phosphate (G3P) produces lyso-phosphatidic acid (LPA) by the catalysis of G3P acyltransferase (GPAT). Another acyl moiety transfer to position 2 of LPA results in the formation of phosphatidic acid (PA), which is catalysed by LPA acyltransferase (LPAAT). Formation of DAG from dephosphorylation of PA is facilitated by phosphatidic acid phosphatase (PAP), and the final transfer of a third acyl chain to position 3 of DAG is acomplished by diacylglycerol acyltransferase (DGAT), leading to synthesis of a neutral TAG. In the present NGS analysis, a single gene was identified for GK, LPAAT, and PAP. Additionally, two and three genes were identified for GPAT and DGAT, respectively. While the transcript levels of GPAT were very similar between macrozooids and haematocysts, those for LPAAT, PAP, and DGAT were expressed at slightly higher levels (~1.5- to 1.9-fold) in the red cysts (Fig. 9). Additionally, TAG can be synthesized through an acyl-CoA-independent pathway mediated by a phospholipid:DAG acyltransferase (PDAT) in microalgae and other eukaryotes. PDAT transfers a fatty acyl group from a phospholipid (PL) or galactolipid to DAG, leading to production of TAG. Additionally, PDAT has been suggested to function as an acyl-CoA-independent diacylglycerol transacylase (DGTA) in *C. reinhardtii,* which produces TAG by using two DAG molecules as both acyl donors and acceptors (Yoon *et al.*, 2012). In the present analysis, a single gene was identified for PDAT, which was slightly up-regulated (1.6-fold) under high irradiance. Early expression analysis (0–24h) via RT–qPCR revealed that the expression of LPAAT was highly up-regulated (10-fold), and that of all other genes except DGAT were moderately up-regulated during 24h under high irradiance (Fig. 9).

Oil globule-associated proteins are known to be involved in various processes, such as globule formation, degradation, stabilization, and in globule–globule interactions or interactions between globules and other organelles (Katz *et al.*, 1995; Frandsen *et al.*, 2001; Murphy, 2001, 2004). Three genes encoding the major oil globule-associated protein, the structural component of the monolayer of oil globules (Peled *et al.*, 2011), did not show any differences between the cells at the two different stages (data not shown). This result implied that the globule-associated protein of this organism might be regulated at the translational or post-translational level to sustain the biogenesis and enormous accumulation of the oil globules.

Although the cellular content of the membrane glycerolipids substantially decreased or remained unchanged in response to elevated light intensity, the transcriptome analysis revealed that a number of genes involved in membrane glycerolipid biosynthesis and turnover were up-regulated at the gene expression level (Supplementary Table S4 available at *JXB* online). Lipidome and transcriptome results taken together allowed for speculation that the *de novo* biosynthesis of the membrane glycerolipids was probably enhanced in response to the increased irradiance and therefore provided newly synthesized membrane glycerolipid molecules with the proper structure and function to replace damaged and degraded lipid species in the cellular membrane or photosynthetic complexes.

### Transcriptome analysis of additional photoprotective mechanisms under high irradiance

Over-reduction of the photosynthetic electron transport chain (PETC) by high irradiance may generate excess ROS, which can damage various cellular components and result in oxidative stress (Apostol *et al.*, 1989; Asada, 1994, 1999; Foyer and Mullineaux, 1994). Previously, Han *et al.* (2012) demonstrated that the capability to prevent ROS accumulation effectively was critical for the survival of *H*. *pluvialis* under high light stress conditions.

Cells can fight against oxidative stress via several defensive mechanisms, including increasing ROS scavenging activity or decreasing ROS production. Scavenging of ROS can be mediated by a variety of enzymes, such as superoxide dismutase (SOD), catalase (CAT), ascorbate peroxidase (APX), glutathione reductase (GR), monodehydroascorbate reductase (MDHAR), dehydroascorbate reductase (DHAR), and glutathione peroxidase (GPX). ROS accumulation can be also counteracted by non-enzymatic, low molecular weight metabolites including ascorbic acid, glutathione (GSH), thioredoxin (TXN), and secondary carotenoids (for reviews, see Blokhina *et al.*, 2003; Moulin *et al.*, 2010). It is possible that the two kinds of antioxidant systems work in concert to protect cells from oxidative damage.

The NGS analysis carried out here identified multiple genes coding for GPX (two), GR (three), and APX (two), and at least one gene for each enzyme was up-regulated by high irradiance, up to ~3-fold (Table 2). As for low molecular antioxidant proteins, only *TXN* genes were detected in the NGS. A single gene was found for DHAR, the transcripts of which were detected only in haematocysts. Two genes identified for *APX* and *GPX* were expressed at a higher level (~1.8- to 3-fold) in haematocysts. All three *GR* genes were up-regulated ~1.6- to 3.8-fold by high irradiation. Four genes were identified for *SOD*, among which one transcript displayed a slight increase (~1.7-fold) in response to high irradiation whereas the other three genes were not affected. Likewise, the transcript levels of a single catalase and *MDHAR* gene were not significantly affected by high light conditions. Based on the present results, it would seem that an increase in the abundance of those transcripts related to ROS scavenging activity could contribute to the protection of cells from oxidative stress induced by high light levels.

The present NGS analysis revealed that high light strongly down-regulated the expression of genes encoding proteins involved in light-harvesting activities, such as the light-harvesting chlorophyll *a*/*b*-binding proteins of photosystems (PS) I and II. High irradiance also decreased the expression of genes coding for PSI and PSII reaction centre proteins (Table 3). The cytochrome *b*_6_ *f* complex functions as a plastoquinine (PQ)-plastocyanin-oxidoreductase to couple PSII and PSI, and is also involved in cyclic electron transport. In the NGS analysis, seven genes that encode the cytochrome *b*_6_ *f* complex were identified and four were down-regulated under high light conditions. Most notably, expression of the most highly active gene in macrozooid cells declined by >30% under high irradiance. The transcript level of an identified plastocyanin gene also decreased by 55% under high light. Among 11 genes encoding oxygen-evolving complexes, the three most highly expressed genes were down-regulated up to ~2-fold in haematocysts, suggesting that high light conditions decreased the primary electron-donating activity from oxygen-evolving proteins. In addition to its role as a cofactor to couple PETC to astaxanthin synthesis, PTOX is known to facilitate reoxidation of the PQ pool in the photosynthetic electron transport chain independently of cytochrome *b*_6_ *f*. PTOX can serve as an alternative electron sink, transferring electrons from the PQ pool to O_2_ to form H_2_O (Cournac *et al.*, 2002; Joet *et al.*, 2002; Peltier and Cournac, 2002). As mentioned earlier, haematocysts displayed higher expression of *PTOX* genes. All of these transcriptomic differentiations that serve to reduce the light-harvesting, reaction centre, and linear electron transport activities may help to relax over-reduced PETC and lead to a decrease in ROS formation. The amounts of ROS produced from late exponential *Haematococcus* macrozooid cells were monitored following exposure to high light conditions for 4 d using H_2_DCFDA. Consistent with the transcriptomic adjustment which was observed, the production of ROS decreased dramatically in cells after 3 d under high irradiance and remained at lower levels thereafter (Supplementary Fig. S8 available at *JXB* online).

**Table 3.** Isotigs of photosystem- and light-harvesting complex-related genes in the *H. pluvialis* transcriptome

Isotig number	Description	Accession	Read ratio (red/green)
isotig04372–04373, 04376–04377	Chloroplast photosystem I reaction centre subunit III (*Chlamydomonas* sp. ICE-L)	ADQ00182.1	0.59
isotig21425–21426	Photosystem I reaction centre, subunit VIII (*Chlamydomonas reinhardtii*)	XP_001703367.1	0.52
isotig09349–09350	Photosystem I reaction centre, subunit VIII (*Chlamydomonas reinhardtii*)	XP_001703367.1	0.60
isotig26971	Photosystem I reaction centre subunit XI (*Chlamydomonas reinhardtii*)	XP_001691084.1	0.63
isotig22073–22074	Photosystem I subunit O (*Chlamydomonas reinhardtii*)	XP_001700109.1	0.69
isotig16438–16439	Photosystem I reaction centre subunit V, chloroplast precursor (*Volvox carteri* f. *nagariensis*)	XP_002949489.1	0.48
isotig12347–12350	Photosystem I reaction centre subunit VI, chloroplast precursor (*Volvox carteri* f. *nagariensis*)	XP_002956888.1	0.52
isotig22915–22916	Photosystem I reaction center subunit psaK, chloroplast precursor (*Volvox carteri* f. *nagariensis*)	XP_002946059.1	0.50
isotig28347	Photosystem I 8.1kDa reaction centre subunit IV (*Chlamydomonas reinhardtii*)	XP_001702611.1	0.55
isotig09222,09224	Photosystem I 8.1kDa reaction centre subunit IV (*Chlamydomonas reinhardtii*)	XP_001702611.1	0.49
isotig22287–22288	Photosystem I 8.1kDa reaction centre subunit IV (*Chlamydomonas reinhardtii*)	XP_001702611.1	0.65
isotig39653	Photosystem I 8.1kDa reaction centre subunit IV (*Chlamydomonas reinhardtii*)	XP_001702611.1	0.41
isotig30606	Photosystem I 8.1kDa reaction centre subunit IV (*Chlamydomonas reinhardtii*)	XP_001702611.1	0.69
isotig04580–04581, 04583–04586, 04589–04590	10kDa photosystem II polypeptide (*Chlamydomonas reinhardtii*)	XP_001696588.1	0.67
isotig02670	Photosystem II 47kDa protein (*Dunaliella salina*)	YP_005089828.1	0.94
isotig45545	Photosystem II protein I (*Dunaliella salina*)	YP_005089795.1	0.63
isotig35094	Photosystem II protein V (*Dunaliella salina*)	YP_005089824.1	0.95
isotig29750	Photosystem II protein Z (*Dunaliella salina*)	YP_005089815.1	0.95
isotig02655–02657, 02659, 02661, 02668	Photosystem II subunit B (*Dunaliella tertiolecta*)	AAX76829.1	0.96
isotig20097–20098	Photosystem II subunit W, chloroplast precursor (*Volvox carteri* f. *nagariensis*)	XP_002955022.1	0.46
isotig31218	Photosystem II subunit 28 (*Chlamydomonas reinhardtii*)	XP_001690537.1	0.72
isotig09395–09397	Oxygen-evolving enhancer protein 3 (*Chlamydomonas reinhardtii*)	XP_001701331.1	0.77
isotig26863	Photosystem II protein D2 (*Chlamydomonas reinhardtii*)	1209190A	0.73
isotig03001–03002, 03006–03007, 03011–03012, 03016–03017, 03021–03022, 03026–03027	Major light-harvesting chlorophyll *a*/*b* protein 2.1 (*Dunaliella salina*)	ABD91646.1	0.38
isotig04198–04209	Major light-harvesting chlorophyll *a*/*b* protein 2.1 (*Dunaliella salina*)	ABD91646.1	0.31
isotig13851–13852	Light-harvesting chlorophyll *a*/*b*-binding protein Lhcb5 (*Chlamydomonas incerta*)	ABD37916.1	0.50
isotig19573–19574	Light-harvesting chlorophyll *a*/*b*-binding protein Lhcb5 (*Chlamydomonas incerta*)	ABD37916.1	0.50
isotig05175, 05178, 05180	Light-harvesting chlorophyll *a*/*b*-binding protein Lhca6 (*Chlamydomonas incerta*)	ABD37908.1	0.44
isotig01744–01788	Light-harvesting chlorophyll *a*/*b*-binding protein of photosystem I (*Volvox carteri* f. *nagariensis*)	XP_002957031.1	0.66
isotig05176–05177, 05181–05186	Light-harvesting protein of photosystem I (*Volvox carteri* f. *nagariensis*)	XP_002958611.1	0.43
isotig10497–10500	Light-harvesting protein of photosystem I (*Volvox carteri* f. *nagariensis*)	XP_002957416.1	0.51
isotig11150–11152	Light-harvesting protein of photosystem I (*Volvox carteri* f. *nagariensis*)	XP_002948151.1	0.68
isotig23593	Light-harvesting protein of photosystem I (*Volvox carteri* f. *nagariensis*)	XP_002958611.1	0.24
isotig28923	Light-harvesting protein of photosystem I (*Volvox carteri f. nagariensis*)	XP_002958611.1	0.29
isotig13760	Light-harvesting protein of photosystem I (*Chlamydomonas reinhardtii*)	XP_001692548.1	0.60
isotig13759, 13761	Light-harvesting complex A protein (*Volvox carteri* f. *nagariensis*)	XP_002950368.1	0.60
isotig20625	Light-harvesting complex A (*Volvox carteri* f. *nagariensis*)	XP_002947520.1	0.03
isotig40410	Chloroplast light-harvesting chlorophyll-*a*/*b*-binding protein (*Chlamydomonas incerta*)	ABA01131.1	0.19
isotig22267–22268	Early light-inducible protein (*Chlamydomonas reinhardtii*)	XP_001695978.1	0.87
isotig29810	Early light-inducible protein (*Chlamydomonas reinhardtii*)	XP_001694751.1	0.71
isotig03544,03547	Low molecular mass early light-induced protein (*Volvox carteri* f. *nagariensis*)	XP_002958870.1	0.52
isotig18101–18102	Light-dependent protochlorophyllide reductase (*Chlamydomonas reinhardtii*)	XP_001689464.1	0.47
isotig03028–03029	Chlorophyll *a*/*b*-binding protein (*Chlamydomonas moewusii*)	P22686.1	0.39
isotig21535–21536	Chlorophyll *a*/*b*-binding protein (*Chlamydomonas reinhardtii*)	XP_001699932.1	0.88
isotig06502–06507	Chlorophyll *a*/*b*-binding protein of photosystem II (*Volvox carteri* f. *nagariensis*)	XP_002951424.1	0.56
isotig39761	Major chlorophyll-binding protein (*Dunaliella salina*)	P20865.1	0.10
isotig13853	Minor chlorophyll *a*/*b*-binding protein of photosystem II (*Chlamydomonas reinhardtii*)	XP_001695927.1	0.66
isotig13130–13132	Oxygen-evolving enhancer protein 1 of photosystem II (*Chlamydomonas reinhardtii*)	XP_001694699.1	0.58
isotig19149–19150	Oxygen-evolving enhancer protein 2 (*Volvox carteri* f. *nagariensis*)	XP_002956365.1	0.67
isotig20107–20108	Oxygen-evolving enhancer protein 2 (*Volvox carteri* f. *nagariensis*)	XP_002956365.1	0.40
isotig27041	Oxygen-evolving enhancer protein 1 of photosystem II (*Chlamydomonas reinhardtii*)	XP_001694699.1	0.54
isotig27610	Oxygen-evolving enhancer protein 1 of photosystem II (*Chlamydomonas reinhardtii*)	XP_001694699.1	0.83
isotig27685	Chloroplast oxygen-evolving complex/thylakoid lumenal 25.6kDa protein (*Chlamydomonas incerta*)	ABA01138.1	1.41
isotig28381	Oxygen-evolving enhancer protein 2 (*Volvox carteri* f. *nagariensis*)	XP_002956365.1	0.73
isotig29135	Chloroplast oxygen-evolving protein 3 (*Chlamydomonas incerta*)	ABA01140.1	0.76
isotig29172	Chloroplast oxygen-evolving protein 3 (*Chlamydomonas incerta*)	ABA01140.1	0.98
isotig29506	Oxygen-evolving enhancer protein 2 of photosystem II (*Chlamydomonas reinhardtii*)	XP_001694126.1	1.03
isotig35073	Oxygen-evolving enhancer protein 2 (*Volvox carteri* f. *nagariensis*)	XP_002956365.1	0.00
isotig09313–09316	Plastocyanin (*Scenedesmus obliquus*)	P26956.2	0.59

In summary, the transcriptomic and lipidomic analyses suggested that under high irradiance stress, *H. pluvialis* cells were protected from photooxidative stress by multiple defensive systems. High irradiance induced formation of astaxanthin and TAG, while reducing membrane glycerolipids, especially those glycolipids making up the photosynthetic complexes and chloroplast membrane matrix. The transcripts of genes involved in astaxanthin biosynthesis—including those responsible for supplying the isoprene precursor via the MEP pathway or converting IPP to astaxanthin—were globally up-regulated to a comparable extent. Accumulation of TAG under high irradiance was attributable to the remarkable up-regulation of *de novo* fatty acid biosynthesis at the gene expression level as well as to the moderate elevation of the TAG assembly pathways. Consequently, enhanced astaxanthin and fatty acid biosynthesis consumed excessive electrons from over-reduced PETC under stress. Increasing enzyme-based ROS scavenging activity and reducing light-harvesting capacity, primary photochemical energy production, and linear electron transport (as inferred from the transcriptomic analyses) also augmented the ability of *H. pluvialis* cells to survive under photooxidative stress.

## Supplementary data

Supplementary data are available at *JXB* online.

Figure S1. Minor and trace triacylglycerol (TAG) species in *H. pluvialis*.

Figure S2. Profile and quantification of PG (A), PC (B), DGTS (C), PI (D), and PE (E) in *H. pluvialis*.

Figure S3. The length and coverage of contigs, isotigs, and reads from the *H. pluvialis* transcriptome database.

Figure S4. A detailed analysis of GO terms of the three functional classes of *H. pluvialis*.

Figure S5. Correlation plot of housekeeping genes in *H. pluvialis* transcriptomes.

Figure S6. KEGG pathway analysis of *H. pluvialis* showing differential gene expression (>2-fold) between macrozooids (A) and cysts (B).

Figure S7. Mevalonate (MVA) and 2-C-methyl-d-erythritol 4-phosphate (MEP) pathways in *H*. *pluvialis*.

Figure S8. ROS production of *H. pluvialis*.

Table S1. Abbreviations used in this paper together with their full names.

Table S2. Total carotenoid and chlorophyll content under the different culture conditions.

Table S3. Housekeeping genes in the *H. pluvialis* transcriptome.

Table S4. Membrane glycerolipid biosynthesis-related genes in the *H. pluvialis* transcriptome.

Supplementary Data
