# Supplementary material for: Comparative analyses of lipidomes and transcriptomes reveal a concerted action of multiple defensive systems against photooxidative stress in Haematococcus pluvialis
Source: J Exp Bot. 2014 May 12;65(15):4317–34. doi: 10.1093/jxb/eru206 (PMC4112636; doi:10.1093/jxb/eru206)
Supplement: Supplementary Data [file supp_65_15_4317__index.html]

Comparative analyses of lipidomes and transcriptomes reveal a concerted action of multiple defensive systems against photooxidative stress in Haematococcus pluvialis — Comparative analyses of lipidomes and transcriptomes reveal a concerted action of multiple defensive systems against photooxidative stress in Haematococcus pluvialis — Supplementary Data 

# Comparative analyses of lipidomes and transcriptomes reveal a concerted action of multiple defensive systems against photooxidative stress in *Haematococcus pluvialis*

## Supplementary Data

Data files

**Files in this Data Supplement:**

- Supplementary Data - Supplementary Data
- Supplementary Data - Supplementary Data
