# Supplementary material for: Comparative analyses of lipidomes and transcriptomes reveal a concerted action of multiple defensive systems against photooxidative stress in Haematococcus pluvialis
Source: J Exp Bot. 2014 May 12;65(15):4317–34. doi: 10.1093/jxb/eru206 (PMC4112636; doi:10.1093/jxb/eru206)
Supplement: Supplementary Data [file supp_eru206_jexbot119974_file002.pdf]

Comparative analyses of lipidomes and transcriptomes reveal a concerted action of multiple defensive systems against photooxidative stress in *Haematococcus pluvialis*

Yunho Gwak[1+], Yong-sic Hwang[2+], Baobei Wang[3], Minju Kim[1], Jooyeon Jeong[1], Choul-Gyun Lee[4], Qiang Hu[5], Danxiang Han[3*] and EonSeon Jin[1*]

[1]Department of Life Science, Research Institute for Natural Science, Hanyang University, Seoul, 133-791, South Korea

[2]Department of Bioscience and Biotechnology, Konkuk University, Seoul, 143-701, South Korea

[3]College of Technology and Innovation, Arizona State University, Mesa, Arizona 85212, USA

[4]Department of Biotechnology, Institute of Industrial Biotechnology, Inha University, Incheon, 402-751, South Korea

[5]Center for Microalgal Biotechnology and Biofuels, Institute of Hydrobiology, Chinese Academy of Sciences, Wuhan, Hubei 430072, China

+Both authors contributed equally to this work

*** Corresponding authors:**

EonSeon Jin: Tel: +82-2-2220-2561; fax: 82-2-2299-2561; e-mail: esjin@hanyang.ac.kr

Danxiang Han: Tel: +1-480-727-5661; email: Danxiang.Han@asu.edu

Fig. 5. Kyoto Encyclopedia of Genes and Genomes (KEGG) pathway analysis of *H. pluvialis* transcripts.

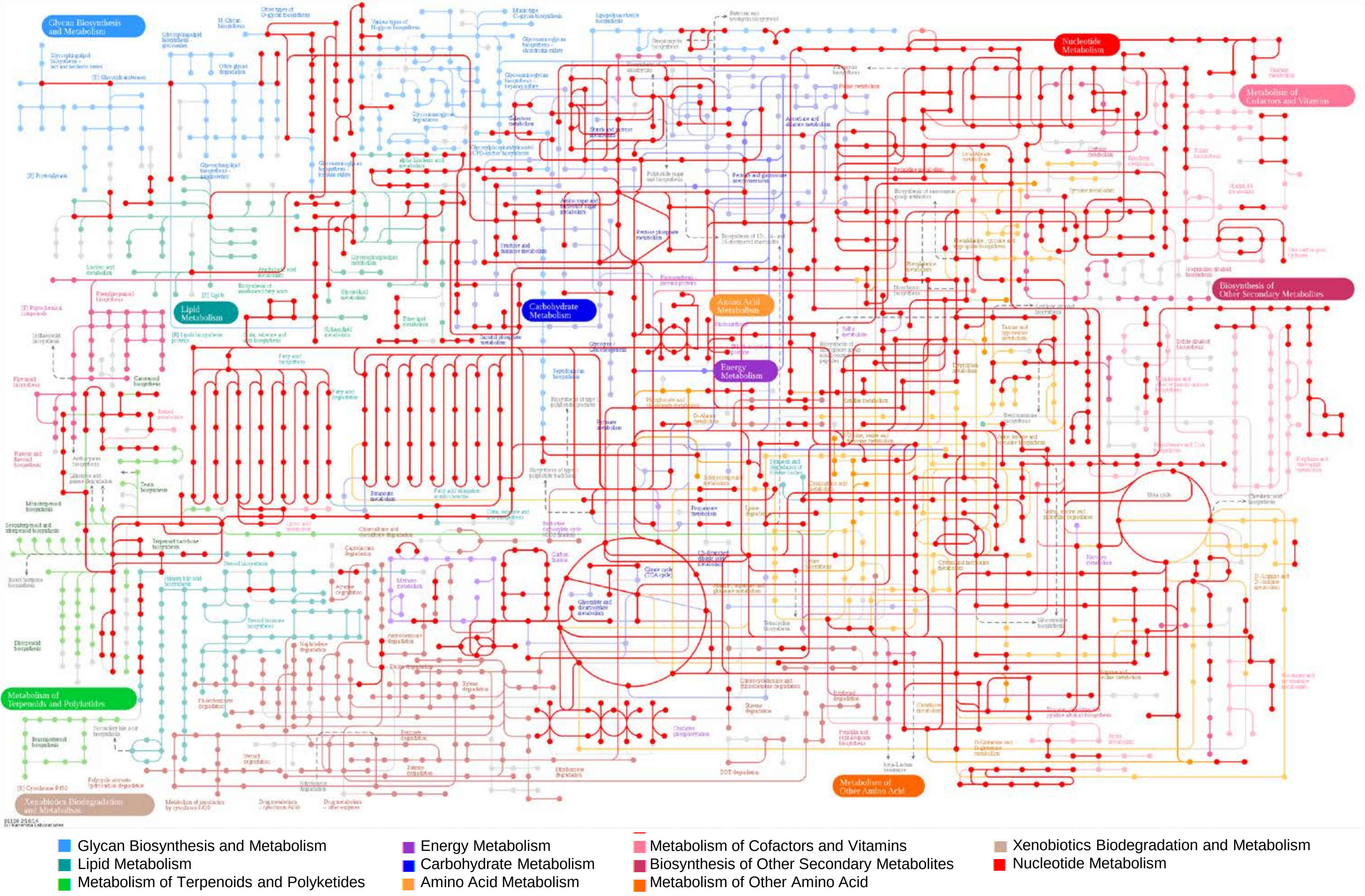

Fig. S6. KEGG pathway analysis of *H. pluvialis*. Macrozooids (A)

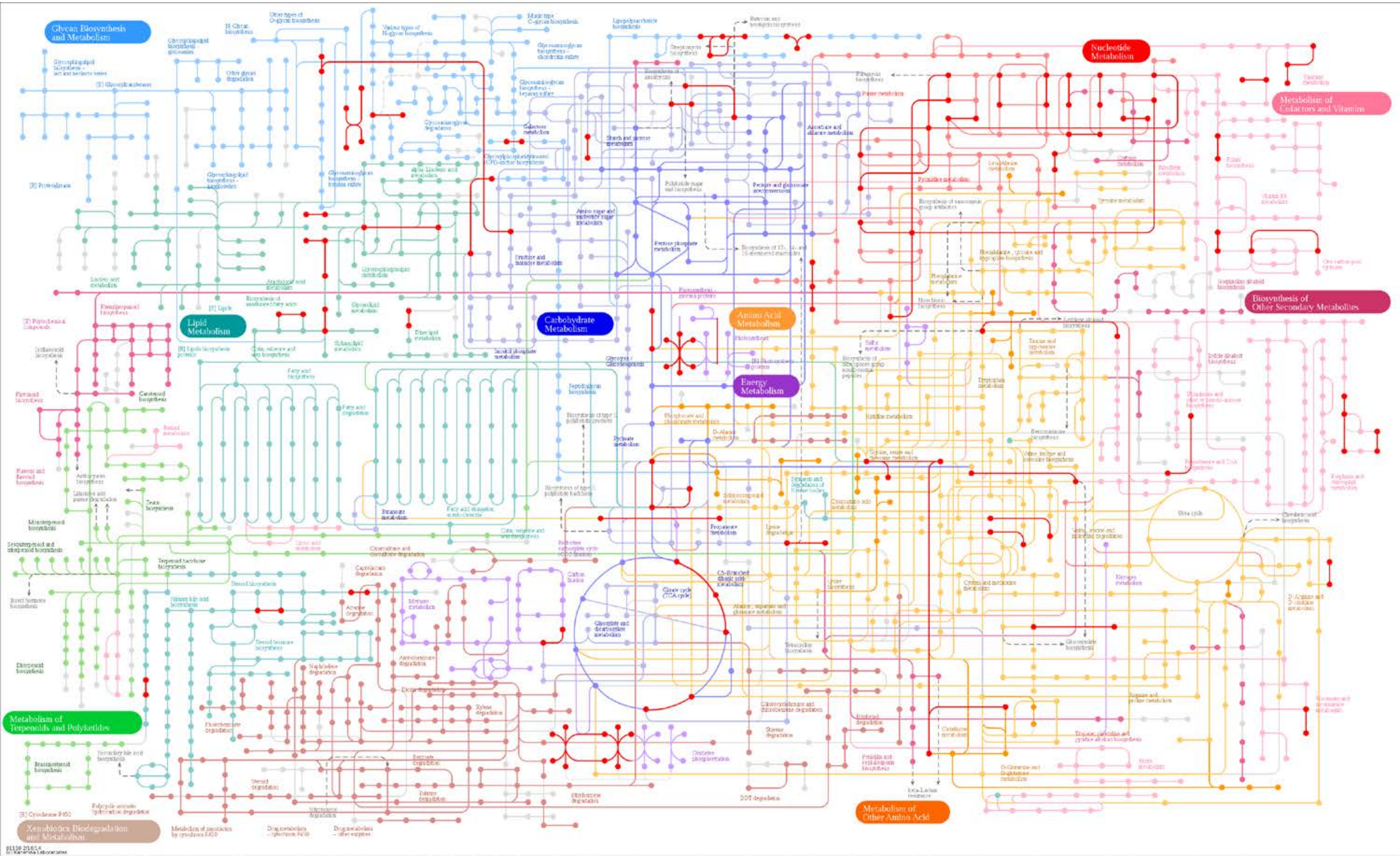

- Glycan Biosynthesis and Metabolism
- Lipid Metabolism
- Metabolism of Terpenoids and Polyketides
- Energy Metabolism
- Carbohydrate Metabolism
- Amino Acid Metabolism
- Metabolism of Cofactors and Vitamins
- Biosynthesis of Other Secondary Metabolites
- Metabolism of Other Amino Acid
- Xenobiotics Biodegradation and Metabolism
- Nucleotide Metabolism

Fig. S6. KEGG pathway analysis of *H. pluvialis*. Cysts (B)

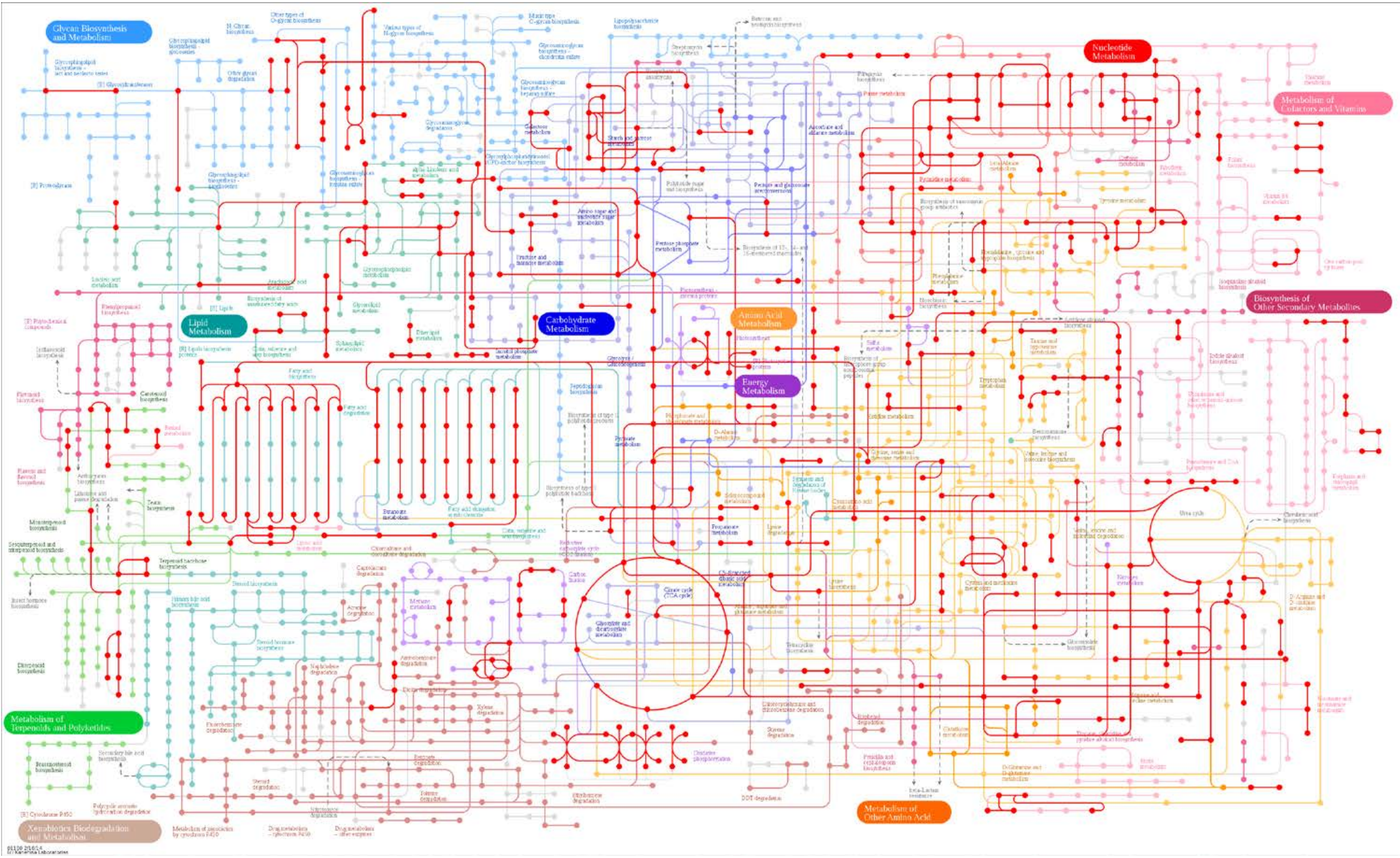

- Glycan Biosynthesis and Metabolism
- Lipid Metabolism
- Metabolism of Terpenoids and Polyketides
- Energy Metabolism
- Carbohydrate Metabolism
- Amino Acid Metabolism
- Metabolism of Cofactors and Vitamins
- Biosynthesis of Other Secondary Metabolites
- Metabolism of Other Amino Acid
- Xenobiotics Biodegradation and Metabolism
- Nucleotide Metabolism
