# Supplementary material for: Comparative analyses of lipidomes and transcriptomes reveal a concerted action of multiple defensive systems against photooxidative stress in Haematococcus pluvialis
Source: J Exp Bot. 2014 May 12;65(15):4317–34. doi: 10.1093/jxb/eru206 (PMC4112636; doi:10.1093/jxb/eru206)
Supplement: Supplementary Data [file supp_eru206_jexbot119974_file001.pdf]

Comparative analyses of lipidomes and transcriptomes reveal a concerted action of multiple defensive systems against photooxidative stress in *Haematococcus pluvialis*

Yunho Gwak[1+], Yong-sic Hwang[2+], Baobei Wang[3], Minju Kim[1], Jooyeon Jeong[1], Choul-Gyun Lee[4], Qiang Hu[5], Danxiang Han[3*] and EonSeon Jin[1*]

[1]Department of Life Science, Research Institute for Natural Science, Hanyang University, Seoul, 133-791, South Korea

[2]Department of Bioscience and Biotechnology, Konkuk University, Seoul, 143-701, South Korea

[3]College of Technology and Innovation, Arizona State University, Mesa, Arizona 85212, USA

[4]Department of Biotechnology, Institute of Industrial Biotechnology, Inha University, Incheon, 402-751, South Korea

[5]Center for Microalgal Biotechnology and Biofuels, Institute of Hydrobiology, Chinese Academy of Sciences, Wuhan, Hubei 430072, China

+Both authors contributed equally to this work

*** Corresponding authors:**

EonSeon Jin: Tel: +82-2-2220-2561; fax: 82-2-2299-2561; e-mail: esjin@hanyang.ac.kr

Danxiang Han: Tel: +1-480-727-5661; email: Danxiang.Han@asu.edu

Supplementary data

Supplementary tables

Table S1-1. Abbreviations and their full names which are used in this paper.

Abbreviations	Full name
ACAT	Acetyl-CoA C-acetyltransferase
ACCase	Acetyl-CoA carboxylase
ACP	Acyl carrier protein
ACS	Acyl CoA synthethase
APX	Ascorbate peroxidase
BKT	β-carotene ketolase
CAT	Catalase
CHY	β-carotene hydroxylase
DAG	Diacylglycerol
DGAT	Diacylglycerol acyltransferase
DGDG	Digalactosyldiacylglycerol
DGTA	Diacylglycerol transacylase
DGTS	Diacylglyceryltrimethylhomo-Ser
DHAR	Dehydroascorbate reductase
DMAPP	Dimethylallyl diphosphate
DXR	1-deoxy-D-xylulose 5-phosphate reductoisomerase
DXS	1-deoxy-D-xylulose-5-phosphate synthase
ENR	Enoyl-AC reductase
FAT	Acyl-ACP thioesterase
FPPS	Farnesyl diphosphate synthase
G3P	Glycerol-3-phosphate
GGPP	Geranylgeranyl pyrophosphate
GGPS	Geranylgeranyl diphosphate synthase
GK	Glycerol kinase
GO	Gene ontology
GPAT	G3P acyltransferase
GPX	Glutathione peroxidase
GR	Glutathione reductase
GSH	Glutathione
HD	3-hydroxyacyl-ACP dehydratase
HDR	4-hydroxy-3-methylbut-2-enyl diphosphate reductase
HDS	1-hydroxy-2-methyl-2-(E)-butenyl-4-diphosphate synthase
HMGS	Hydroxymethylglutaryl-CoA
HPLC	High-performance liquid chromatography

Table S1-2. Abbreviations and their full names which are used in this paper.

Abbreviations	Full name
IPP	Isopentenyl diphosphate
IPPI	Isopentenyl diphosphate isomerase
KAR	3-ketoacyl-ACP-reductase
KAS	3-ketoacyl-ACP-synthase
KEGG	Kyoto encyclopedia of genes and genomes
KO	KEGG orthology
LPA	Lyso-phophatidic acid
LPAAT	LPA acyltransferase
MCAT	Malonyl-CoA:ACP transacylase
MDHAR	Monodehydroascorbate reductase
MEP pathway	2-C-methyl-D-erythritol 4-phosphate pathw
MGDG	Monogalactosyldiacylglycerol
MVA pathway	Mevalonate pathway
NGS	Next generation sequencing
PA	Phosphatidic acid
PAP	Phosphatidic acid phosphatase
PC	Phosphatidylcholine
PDAT	Phospholipid:DAG acyltransferase
PDS	Phytoene desaturase
PE	Phosphatidylethanolamine
PETC	Photosynthetic electron transport chain
PG	Phosphatidylglycerol
PI	Phosphatidylinositol
PL	Phospholipid
PQ	Plastoquinone
PSY	Phytoene synthase
PTOX	Plastid terminal oxidase
ROS	Reactive oxygen species
SACPD	Stearoyl-ACP desaturase
SOD	Superoxide dismutase
SQDG	Sulfoquinovosyl diacylglycerol
TAG	Triacylglycerols
TXN	Tioredoxin

Table S2. Total carotenoid and chlorophyll content at the different culture conditions.

	Cell density ($cells \cdot mL^{-1}$)	Total carotenoid ($\mu g \cdot mL^{-1}$)	Total chlorophyll ($\mu g \cdot mL^{-1}$)
Initial stage of cells	$0.27 \times 10^5 \pm 0.34$	0.02 ± 0.01	0.10 ± 0.01
Macrozoids (late exponential stage)	$2.23 \times 10^5 \pm 1.26$	1.50 ± 0.02	2.32 ± 0.01
Haematosysts (2 days after HL)	$2.40 \times 10^5 \pm 0.83$	6.22 ± 0.01	1.60 ± 0.03
Haematocysts (4 days after HL)	$2.51 \times 10^5 \pm 0.45$	9.54 ± 0.03	1.48 ± 0.02

Table S3-1. Housekeeping genes in the *H. pluvialis* transcriptome.

**Isogroup**	**Isotiug number**	**Description**	**Accession**	**Read ratio (Red/Green)**
isogroup00137	isotig03365-03400	AF416462_1 actin-like protein [*Chlamydomonas moewusii*]	AAQ03597.1	1.13
isogroup00180	isotig03929-03930	Actin/actin-like protein [*Coccomyxa subellipsoidea* C-169]	EIE26065.1	1.21
isogroup00180	isotig03933-03936	actin-related protein Arp6 [*Volvox carteri f. nagariensis*]	XP_002953065.1	1.28
isogroup01235	isotig10021-10023	actin [*Chlamydomonas reinhardtii*]	XP_001699068.1	1.44
isogroup02007	isotig12785-12787	actin [*Chlamydomonas reinhardtii*]	XP_001699068.1	1.84
isogroup02158	isotig13238-13240	actin-related protein [*Chlamydomonas reinhardtii*]	XP_001692106.1	1.07
isogroup03128	isotig15576-15577	actin-related protein Arp3 [*Volvox carteri f. nagariensis*]	XP_002949421.1	0.76
isogroup05043	isogroup05043	actin-related protein [*Chlamydomonas reinhardtii*]	XP_001694089.1	4.75
isogroup05588	isotig20464	actin II [*Telotrochidium matiense*]	AAM77135.1	1.58
isogroup06946	isotig23179	Actin/actin-like protein [*Coccomyxa subellipsoidea* C-169]	EIE26065.1	1.58
isogroup06946	isotig23180	actin-related protein Arp6 [*Volvox carteri f. nagariensis*]	XP_002953065.1	1.58
isogroup12428	isotig29040	actin-related protein ArpC4 [*Volvox carteri f. nagariensis*]	XP_002945817.1	2.06
isogroup14906	isotig31518	AF416462_1 actin-like protein [*Chlamydomonas moewusii*]	AAQ03597.1	2.85
isogroup00052	isotig01636-01684	alpha-tubulin [*Haematococcus pluvialis*]	AAX63396.1	0.60
isogroup00056	isotig01833-01844	beta tubulin [*Chlamydomonas incerta*]	O04386.1	0.62
isogroup00056	isotig01845-01856	beta tubulin [*Volvox carteri f. nagariensis*]	XP_002953617.1	0.66
isogroup00066	isotig02116, 02117, 02130, 02132, 02141, 02141, 02146, 02148, 02150, 02151	beta tubulin [*Chlamydomonas incerta*]	O04386.1	0.54
isogroup00066	isotig02111, 02113, 02119, 02121, 02123, 02127, 02129, 02134, 02136, 02139	beta tubulin [*Volvox carteri f. nagariensis*]	XP_002953617.1	0.56
isogroup00066	isotig02112, 02114, 02115, 02118, 02120, 02122, 02124-02126, 02128, 02131, 02133, 02135, 02137, 02138, 02140, 02143-02145, 02147, 02149, 02152-02155	tubulin beta chain [*Cryptosporidium parvum Iowa II*]	XP_627803.1	0.57
isogroup14032	isotig30644	tubulin Uni3 [*Chlamydomonas reinhardtii*]	AAB71840.1	1.58
isogroup00402	isotig05836-05838	ubiquitin [*Dianthus caryophyllus*]	BAL61088.1	1.04
isogroup03515	isotig16337-16338	E2 ubiquitin-conjugating enzyme [*Chlamydomonas reinhardtii*]	XP_001703521.1	1.42
isogroup04902	isotig19091	PREDICTED: ubiquitin conjugation factor E4 A-like [*Saccoglossus kowalevskii*]	XP_002733556.1	1.00
isogroup05543	isotig20373-20374	ubiquitin conjugating enzyme E2 [*Chlamydomonas reinhardtii*]	XP_001690015.1	0.92
isogroup06010	isotig21307-21308	ubiquitin conjugating enzyme [*Chlamydomonas reinhardtii*]	XP_001689734.1	0.93
isogroup09489	isotig26101	PREDICTED: LOW QUALITY PROTEIN: ubiquitin-60S ribosomal protein L40-like [*Brachypodium distachyon*]	XP_003561800.1	1.33
isogroup13015	isotig29627	Ubiquitin-conjugating enzyme E2 S [*Rana catesbeiana*]	C1C3R6.1	0.71
isogroup06450	isotig22187-22188	small ubiquitin-like modifier 2 [*Chlamydomonas reinhardtii*]	XP_001695782.1	1.29
isogroup11292	isotig27904	small ubiquitin-like modifier 1 [*Chlamydomonas reinhardtii*]	XP_001695783.1	0.51
isogroup14914	isotig31526	small ubiquitin-like modifier 2 [*Chlamydomonas reinhardtii*]	XP_001695782.1	1.18
isogroup00001	contig00258	eukaryotic translation elongation factor 1 alpha 1 [*Chlamydomonas reinhardtii*]	XP_001696568.1	1.65
isogroup00131	isotig03317-03318	elongation factor-1 alpha-like protein [*Gonium pectorale*]	BAF99250.1	1.48
isogroup00360	isotig05597-05602	elongation factor 2 [*Chlamydomonas reinhardtii*]	XP_001703215.1	1.76
isogroup00675	isotig07555-07556	elongation factor-1 alpha-like protein [*Gonium pectorale*]	BAF99250.1	1.43
isogroup01142	isotig09649-09652	elongation factor-like protein [*Volvox carteri f. nagariensis*]	XP_002958738.1	1.02
isogroup02653	isotig14629-14630	elongation factor EF-Tu-like protein [*Chlamydomonas reinhardtii*]	XP_001699617.1	1.89
isogroup09537	isotig26149	elongation factor-like protein [*Chlamydomonas reinhardtii*]	XP_001699137.1	2.37
isogroup10338	isotig26950	elongation factor 1-alpha, putative [*Ricinus communis*]	XP_002518073.1	2.58
isogroup10956	isotig27568	translational elongation factor Tu [*Chlamydomonas moewusii*]	ABU88343.1	1.64
isogroup13009	isotig29621	elongation factor-like protein [*Chlamydomonas reinhardtii*]	XP_001699137.1	4.27
isogroup15403	isotig32015	elongation factor 2 [*Parachlorella kessleri*]	P28996.1	3.32
isogroup18882	isotig35494	translation elongation factor EF-1, subunit alpha [*Vulcanisaeta distributa* DSM 14429]	YP_003901148.1	4.75

Table S3-2. Housekeeping genes in the *H. pluvialis* transcriptome.

Isogroup	Isotiug number	Description	Accession	Read ratio (Red/Green)
isogroup10661	isotig27273	phosphoglycerate mutase [*Chlamydomonas reinhardtii*]	XP_001701935.1	1.04
isogroup12479	isotig29091	phosphoglycerate mutase [*Chlamydomonas reinhardtii*]	XP_001702221.1	0.95
isogroup01467	isotig10867-10869	phosphoglycerate mutase [*Volvox carteri f. nagariensis*]	XP_002947424.1	1.09
isogroup02719	isotig14761-14762	phosphoglycerate mutase [*Chlamydomonas reinhardtii*]	XP_001703522.1	0.90
isogroup04022	isotig17331-17332	phosphoglycerate mutase [*Chlamydomonas reinhardtii*]	XP_001696212.1	2.17
isogroup06559	isotig22405-22406	phosphoglycerate mutase [*Chlamydomonas reinhardtii*]	XP_001698403.1	1.42
isogroup03238	isotig15793-15794	60S ribosomal protein L11 [*Elaeis guineensis*]	ACF06486.1	0.90
isogroup10723	isotig27335	60S ribosomal protein L18a-2 [*Coccomyxa subellipsoidea* C-169]	EIE27736.1	0.80
isogroup06230	isotig21747-21748	60S ribosomal protein L18a-2 [*Coccomyxa subellipsoidea* C-169]	EIE27736.1	1.22
isogroup09489	isotig26101	PREDICTED: LOW QUALITY PROTEIN: ubiquitin-60S ribosomal protein L40-like [*Brachypodium distachyon*]	XP_003561800.1	1.33
isogroup17490	isotig34102	60S ribosomal protein L30 [*Loa loa*]	XP_003141401.1	0.88
isogroup02469	isotig14168-14169	60S acidic ribosomal protein P2, putative [*Aspergillus clavatus* NRRL 1]	XP_001275311.1	0.66
isogroup03516	isotig16339-16340	60S acidic ribosomal-like protein P2 [*Phragmatopoma lapidosa*]	AEF59082.1	0.90
isogroup03407	isotig16127-6128	40S ribosomal protein S5 [*Chlamydomonas* sp. ICE-L]	CAJ45370.1	1.31
isogroup06284	isotig21855-21856	plastid/chloroplast ribosomal protein L19 [*Volvox carteri f. nagariensis*]	XP_002956886.1	1.61
isogroup11516	isotig28128	mitochondrial ribosomal protein L19 [*Chlamydomonas reinhardtii*]	XP_001700862.1	2.41
isogroup03415	isotig16143-16144	ribosomal protein L32 [*Chlamydomonas reinhardtii*]	XP_001695517.1	1.12
isogroup00131	isotig03315-03316	eukaryotic translation initiation factor 1 alpha 1 [*Volvox carteri f. nagariensis*]	XP_002945791.1	1.48
isogroup07525	isotig24137	eukaryotic translation initiation factor 3c [*Volvox carteri f. nagariensis*]	XP_002947123.1	1.94
isogroup10009	isotig26621	eukaryotic translation initiation factor 3f [*Volvox carteri f. nagariensis*]	XP_002950817.1	1.35
isogroup10956	isotig27568	translational elongation factor Tu [*Chlamydomonas moewusii*]	ABU88343.1	1.64
isogroup00721	isotig07752-07755	malate dehydrogenase [*Volvox carteri f. nagariensis*]	XP_002954828.1	2.70
isogroup02938	isotig15198-15199	malate dehydrogenase [*Chlamydomonas reinhardtii*]	XP_001693118.1	2.10
isogroup08260	isotig24872	malate dehydrogenase, cytoplasmic [*Chlorella variabilis*]	EFN52647.1	1.28
isogroup10899	isotig27511	malate dehydrogenase [*Chlamydomonas reinhardtii*]	XP_001703167.1	0.84
isogroup11265	isotig27877	malate dehydrogenase [*Chlamydomonas reinhardtii*]	XP_001703167.1	1.16
isogroup00545	isotig06783	mitogen activated protein kinase kinase kinase 11 [*Chlamydomonas reinhardtii*]	XP_001699990.1	1.54
isogroup02723	isotig14769-14770	mitogen activated protein kinase kinase kinase 11 [*Chlamydomonas reinhardtii*]	XP_001699990.1	0.24
isogroup04451	isotig18189-18190	mitogen activated protein kinase kinase kinase 11 [*Chlamydomonas reinhardtii*]	XP_001699990.1	1.34
isogroup11100	isotig27712	mitogen activated protein kinase kinase kinase 11 [*Chlamydomonas reinhardtii*]	XP_001699990.1	1.90
isogroup16395	isotig33007	mitogen activated protein kinase kinase kinase 11 [*Chlamydomonas reinhardtii*]	XP_001699990.1	0.95
isogroup04790	isotig18867	cyclophilin-like protein [*Coccomyxa subellipsoidea* C-169]	EIE24486.1	1.54
isogroup05066	isotig19420	cyclophilin-like protein [*Chlamydomonas reinhardtii*]	XP_001701607.1	0.61
isogroup13056	isotig29668	cyclophilin-like protein [*Chlamydomonas reinhardtii*]	XP_001701608.1	4.27
isogroup00452	isotig06258-06259	phosphoglycerate kinase [*Chlamydomonas reinhardtii*]	XP_001699523.1	0.7
isogroup02274	isotig13585	phosphoglycerate kinase [*Chlamydomonas reinhardtii*]	XP_001702049.1	1.2
isogroup09035	isotig25647	phosphoglycerate kinase [*Chlamydomonas reinhardtii*]	XP_001699523.1	0.43
isogroup01257	isotig10109-10112	glyceraldehyde 3-phosphate dehydrogenase [*Chlamydomonas reinhardtii*]	XP_001702068.1	1.12
isogroup03130	isotig15580-15581	chloroplast glyceraldehyde-3-phosphate dehydrogenase [*Dunaliella viridis*]	ABC00187.1	1.08
isogroup04914	isotig19115-19116	chloroplast glyceraldehyde-3-phosphate dehydrogenase [*Dunaliella viridis*]	ABC00187.1	0.95
isogroup04535	isotig18358	tetratricopeptide repeat protein [*Myxococcus xanthus* DK 1622]	YP_629366.1	5.71
isogroup14465	isotig31077	tetratricopeptide repeat protein, circadian expression [*Chlamydomonas reinhardtii*]	XP_001699965.1	1.04
isogroup15560	isotig32172	tetratricopeptide repeat protein, circadian expression [*Chlamydomonas reinhardtii*]	XP_001699965.1	5.71
isogroup02916	isotig15154	glycogen synthase kinase 3 [*Chlamydomonas reinhardtii*]	XP_001690881.1	2.29
isogroup08750	isotig25362	glycogen synthase kinase 3 [*Chlamydomonas reinhardtii*]	XP_001690881.1	0.97
isogroup06954	isotig23195	polyadenylate binding protein [*Coccomyxa subellipsoidea* C-169]	EIE25350.1	0.55
isogroup18104	isotig34716	polyadenylate-binding protein, putative [*Ricinus communis*]	XP_002511041.1	1.90
isogroup09130	isotig25742	protein disulfide isomerase [*Chlamydomonas reinhardtii*]	XP_001699770.1	0.87
isogroup15365	isotig31977	protein disulfide isomerase [*Chlamydomonas reinhardtii*]	XP_001699351.1	1.04
isogroup15902	isotig32514	PREDICTED: DNA-directed RNA polymerases I, II, and III subunit RPABC5-like [*Glycine max*]	XP_003526546.1	1.24
isogroup16107	isotig32719	PREDICTED: DNA-directed RNA polymerases I, II, and III subunit RPABC5-like [*Glycine max*]	XP_003526546.1	1.99

Table S3-3. Housekeeping genes in the *H. pluvialis* transcriptome.

Isogroup	Isotiug number	Description	Accession	Read ratio (Red/Green)
isogroup09735	isotig26347	adenosine deaminase [*Acinetobacter* sp. P8-3-8]	ZP_09141828.1	1.22
isogroup02873	isotig15068-15069	lysyl-trna synthetase, LysRS [*Chlamydomonas reinhardtii*]	XP_001697493.1	2.35
isogroup16588	isotig33200	6-phosphogluconate dehydrogenase, decarboxylating [*Chlamydomonas reinhardtii*]	XP_001696880.1	1.42
isogroup01651	isotig11444-11447	hexokinase [*Volvox carteri f. nagariensis*]	XP_002956604.1	2.20
isogroup00236	isotig04487-4498	glucose-6-phosphate isomerase [*Dunaliella salina*]	ACI29026.1	1.36
isogroup01284	isotig10217-10220	enolase [*Dunaliella salina*]	AAO86694.1	2.08
isogroup04820	isotig18927-18928	mitochondrial cytochrome c oxidase subunit 4, 13 kD [*Chlamydomonas reinhardtii*]	XP_001693699.1	1.38
isogroup03416	isotig16145-16146	TPA_inf: mitochondrial ATP synthase subunit delta [*Polytomella parva*]	ADE92943.1	1.09
isogroup02249	isotig13510-13511	20S proteasome beta subunit, type 4 [*Chlamydomonas reinhardtii*]	XP_001695438.1	1.11
isogroup11159	isotig27771	ADP-ribosylation factor 1 [*Coccomyxa subellipsoidea* C-169]	EIE19146.1	1.14
isogroup02140	isotig13184	cytidine deaminase [*Volvox carteri f. nagariensis*]	XP_002949245.1	0.91
isogroup04756	isotig18799-18800	spermidine synthase [*Chlamydomonas reinhardtii*]	XP_001702843.1	2.25
isogroup01256	isotig10105-10108	serine/threonine protein kinase 19 [*Chlamydomonas reinhardtii*]	XP_001690043.1	1.76
isogroup01442	isotig10792-10794	succinate dehydrogenase subunit A, mitochondrial [*Volvox carteri f. nagariensis*]	XP_002949632.1	1.14
isogroup04881	isotig19049-19050	small nuclear ribonucleoprotein Sm D2 [*Chlamydomonas reinhardtii*]	XP_001698348.1	1.12

Table S4. Membrane glycerolipid biosynthesis–related genes in the *H. pluvialis* transcriptome.

Isogroup	Isotig number	Description	Accession	Read ratio (Red/Green)
isogroup04513	isotig18313-18314	UDP-sulfoquinovose synthase [*Chlamydomonas reinhardtii*]	XP_001697898.1	1.99
isogroup00935	Isotig08834-08837	phosphatidate cytidylyltransferase [*Chlamydomonas reinhardtii*]	XP_001691839.1	2.14
isogroup02829	Isotig14980-14981	phosphatidate cytidylyltransferase [*Chlamydomonas reinhardtii*]	XP_001691107.1	1.32
isogroup05358	Isotig20003-20004	phosphatidylglycerophosphate synthase [*Chlamydomonas reinhardtii*]	XP_001701223.1	1.58
isogroup12714	isotig29326	phosphatidylglycerolphosphate synthase 2 [*Arabidopsis thaliana*]	NP_191063.1	1.58
isogroup04232	Isotig117751-7752	membrane bound O-acyl transferase family protein [*Coccomyxa subellipsoidea* C-169]	EIE25092.1	2.57
isogroup08206	isotig24818	membrane bound O-acyl transferase family protein [*Coccomyxa subellipsoidea* C-169]	EIE25092.1	0.75
isogroup12281	isotig28893	phospholipase A2 [*Chlamydomonas reinhardtii*]	XP_001699857.1	1.66
isogroup03847	Isotig16981-16982	phosphoethanolamine N-methyltransferase [*Spinacia oleracea*]	Q9M571.1	2.02
isogroup04400	Isotig18087-18088	CTP-phosphoethanolamine cytidylyltransferase [*Coccomyxa subellipsoidea* C-169]	XP_005647873.1	1.40
isogroup11956	isotig28568	phosphatidylinositol synthase [*Volvox carteri f. nagariensis*]	XP_002948374.1	2.06

Supplementary figures

Fig. S1.

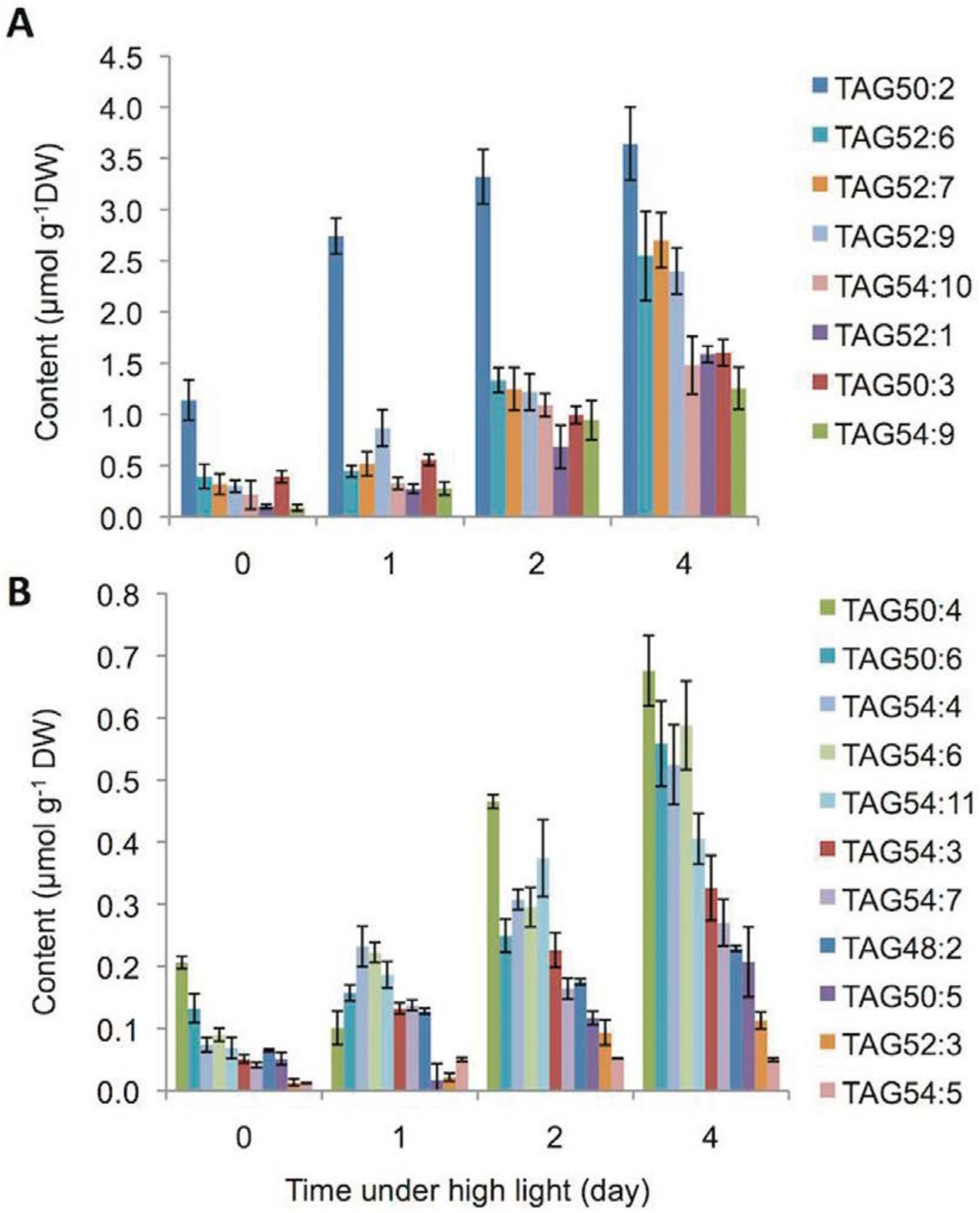

Fig. S1. Minor (A) and trace (B) triacylglycerol (TAG) species in *H. pluvialis*.

Fig. S2.

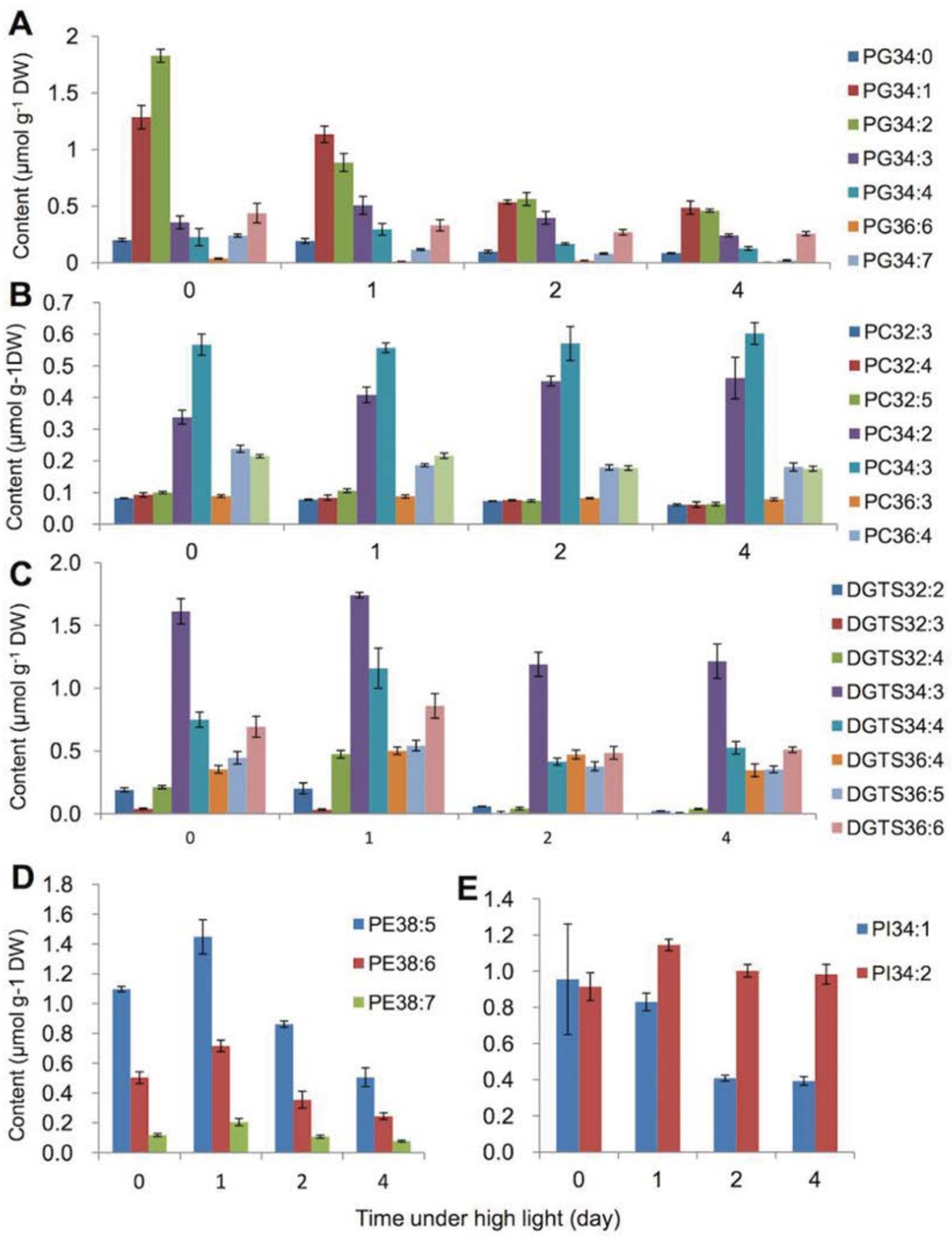

Fig. S2. Profile and quantification of PG (A), PC (B), DGTS (C), PI (D) and PE (E) in *H. pluvialis*. DGTS, Diacylglyceryltrimethylhomo-Ser; PC, Phosphatidylcholine; PE, Phosphatidylethanolamine; PG, Phosphatidylglycerol; PI, Phosphatidylinositol.

Fig. S3.

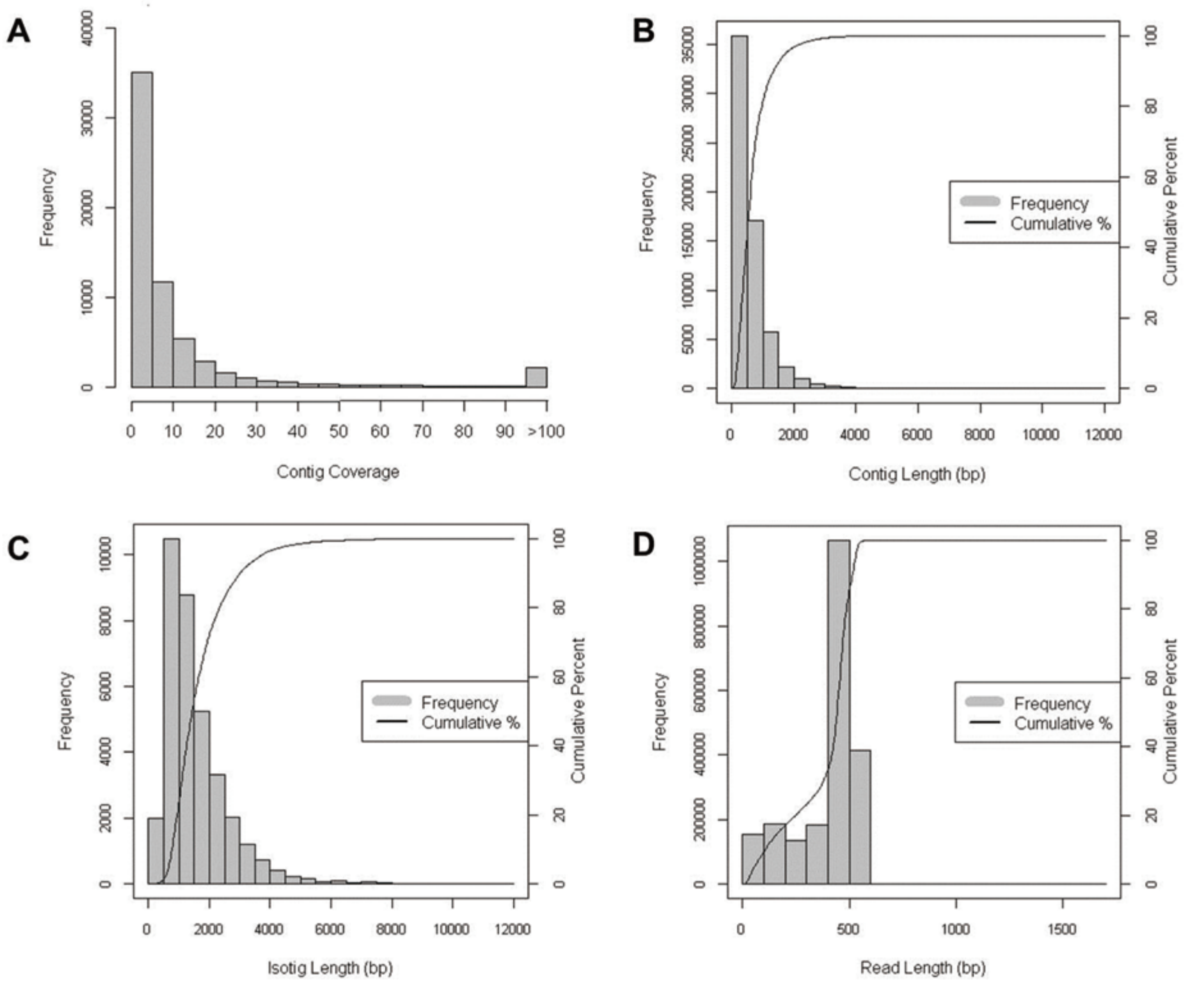

Fig. S3. The length and coverage of contigs, isotigs, and reads from the *H. pluvialis* transcriptome database. Panels A and B represent the contig coverage and length, respectively. Panels C and D indicate the length of isotigs and reads, respectively.

Fig. S4a.

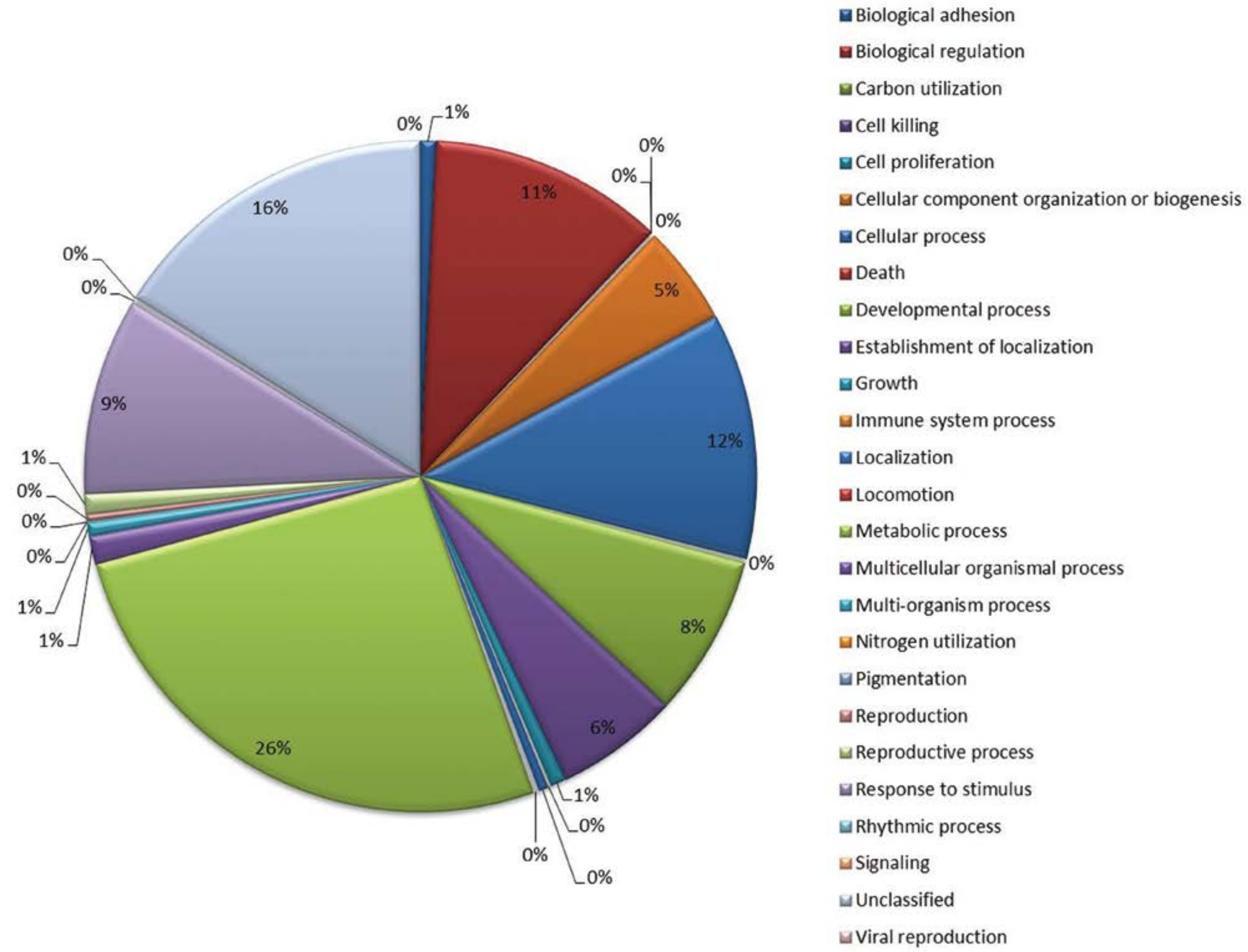
0%
1%
11%
0%
0%
0%
5%
16%
0%
0%
12%
9%
1%
0%
0%
0%
1%
1%
0%
8%
6%
26%
1%
0%
0%
0%
Biological adhesion
Biological regulation
Carbon utilization
Cell killing
Cell proliferation
Cellular component organization or biogenesis
Cellular process
Death
Developmental process
Establishment of localization
Growth
Immune system process
Localization
Locomotion
Metabolic process
Multicellular organismal process
Multi-organism process
Nitrogen utilization
Pigmentation
Reproduction
Reproductive process
Response to stimulus
Rhythmic process
Signaling
Unclassified
Viral reproduction

Fig. S4b.

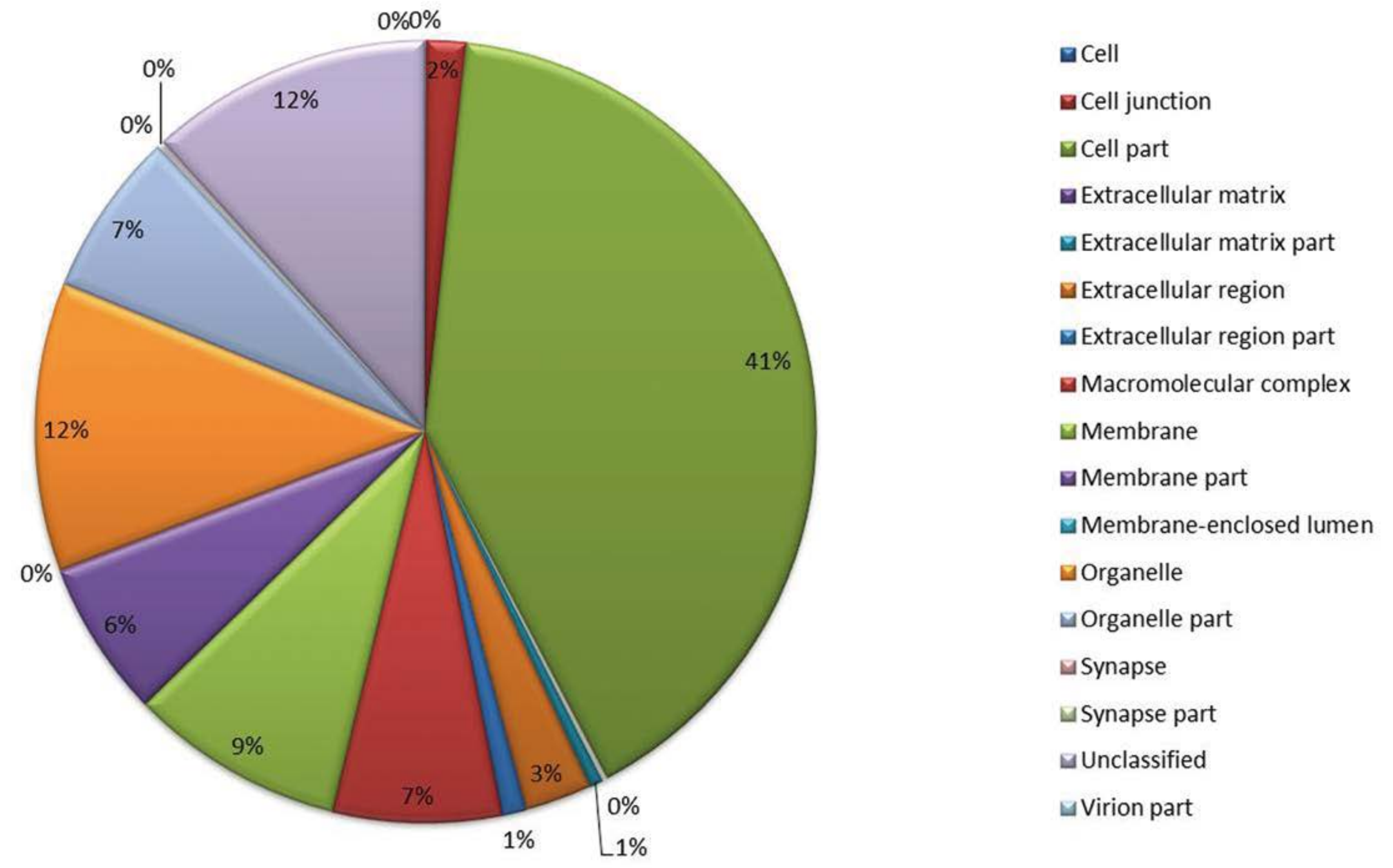
0%0%
2%
41%
0%
0%
12%
7%
12%
0%
6%
9%
7%
1%
3%
0%
1%
Cell
Cell junction
Cell part
Extracellular matrix
Extracellular matrix part
Extracellular region
Extracellular region part
Macromolecular complex
Membrane
Membrane part
Membrane-enclosed lumen
Organelle
Organelle part
Synapse
Synapse part
Unclassified
Virion part

Fig. S4c.

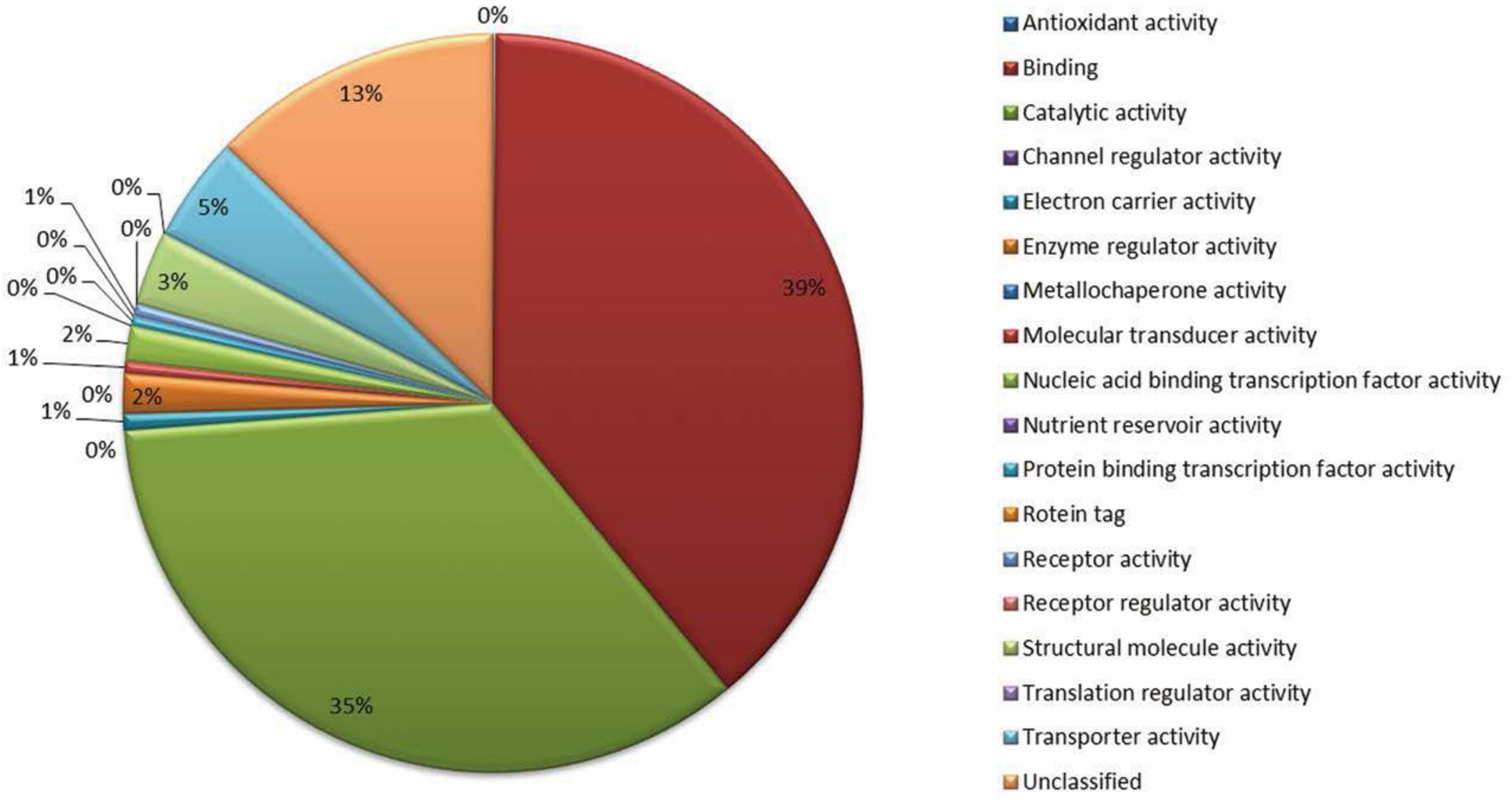

Fig. S4. A detailed analysis of GO terms of the three functional classes of *H. pluvialis*. The total of 61 GO terms were assigned to 22,431 cDNAs (including isotigs and contigs). A: biological processes; B: cellular components; C: molecular functions.

Fig. S5.

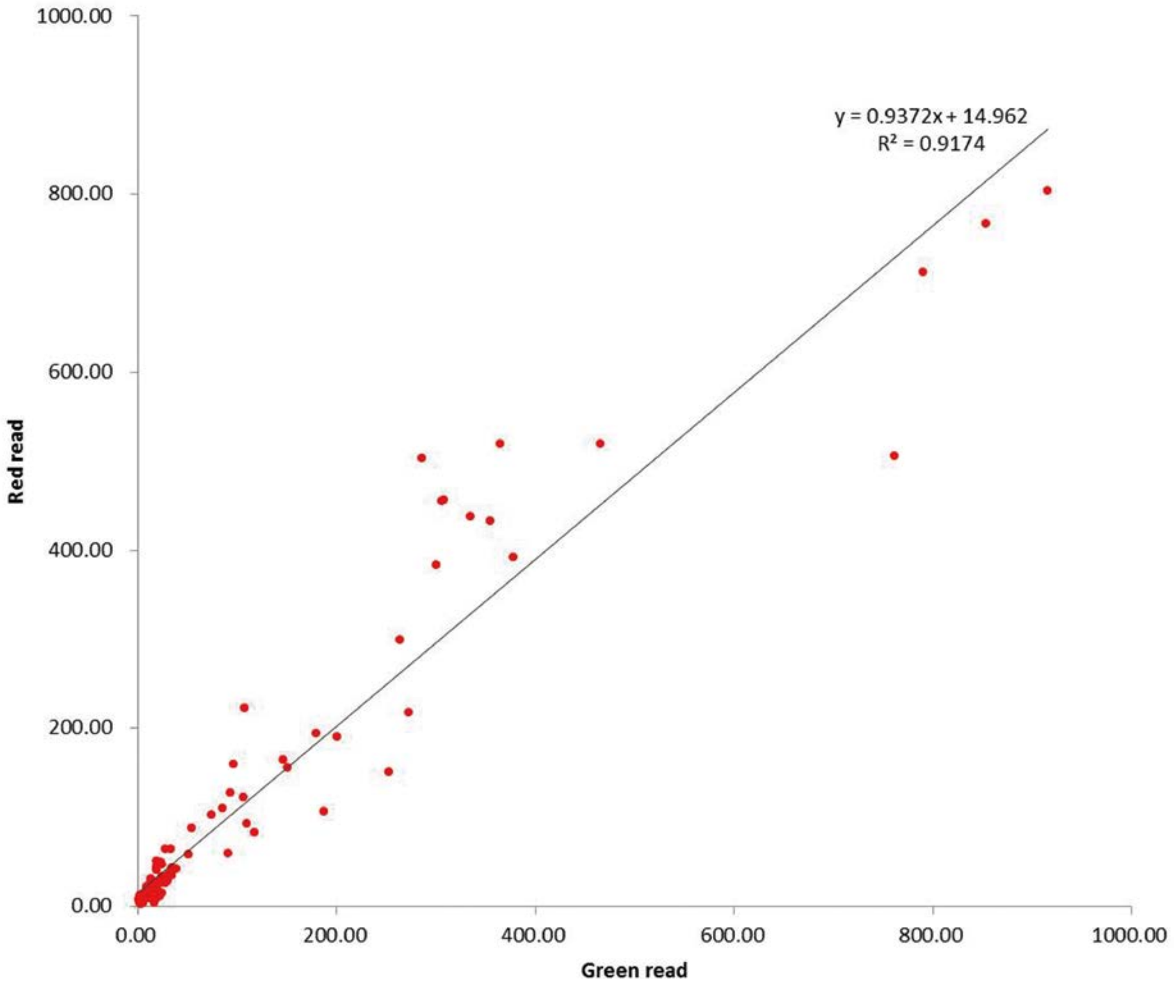

Fig. S5. Correlation plot of housekeeping genes in *H. pluvialis* transcriptomes. The slope value is 0.9372 and the R^2 value is 0.9174.

Fig. S6a.

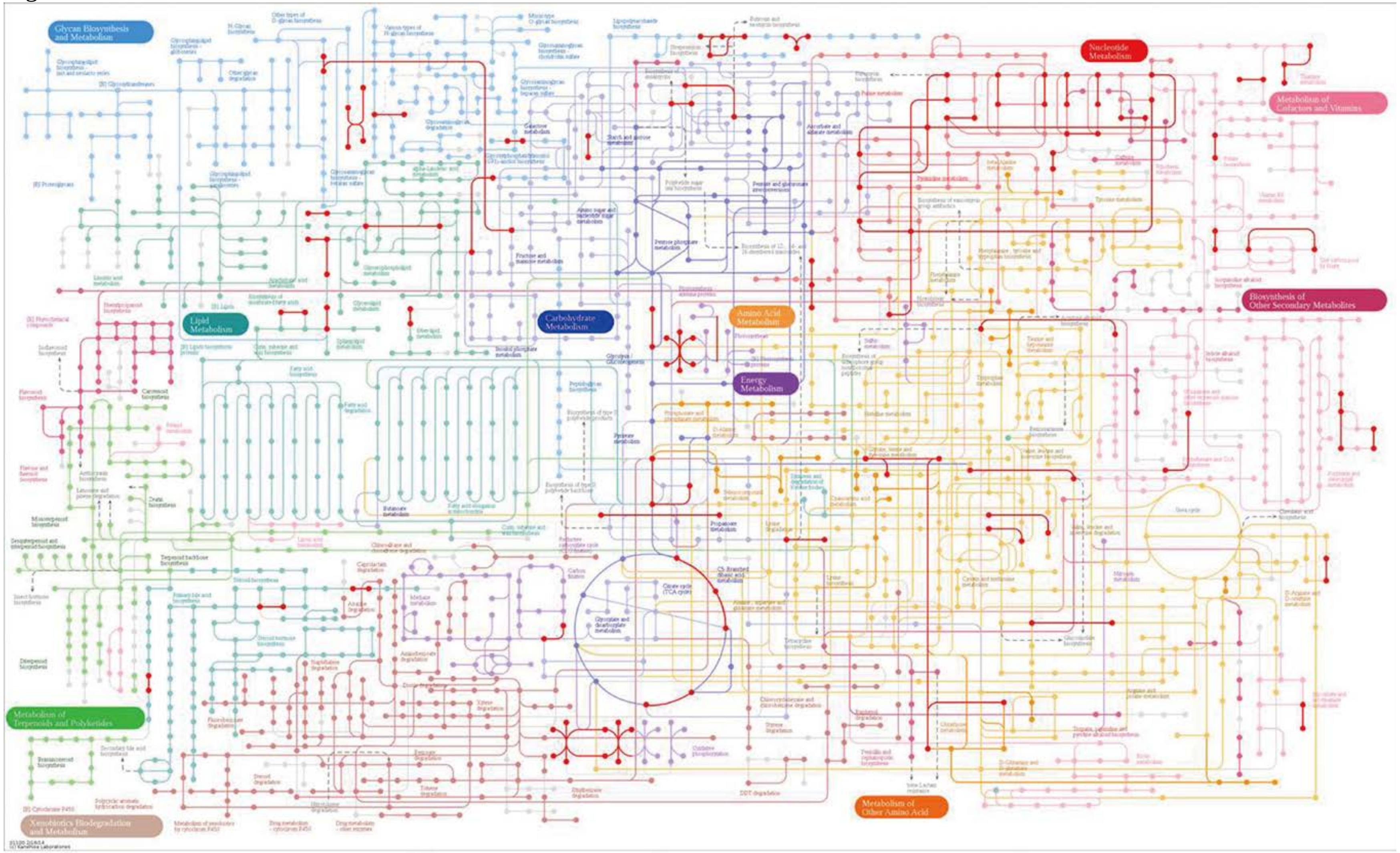

- Glycan Biosynthesis and Metabolism
- Lipid Metabolism
- Metabolism of Terpenoids and Polyketides
- Energy Metabolism
- Carbohydrate Metabolism
- Amino Acid Metabolism
- Metabolism of Cofactors and Vitamins
- Biosynthesis of Other Secondary Metabolites
- Metabolism of Other Amino Acid
- Xenobiotics Biodegradation and Metabolism
- Nucleotide Metabolism

Fig.S6b.

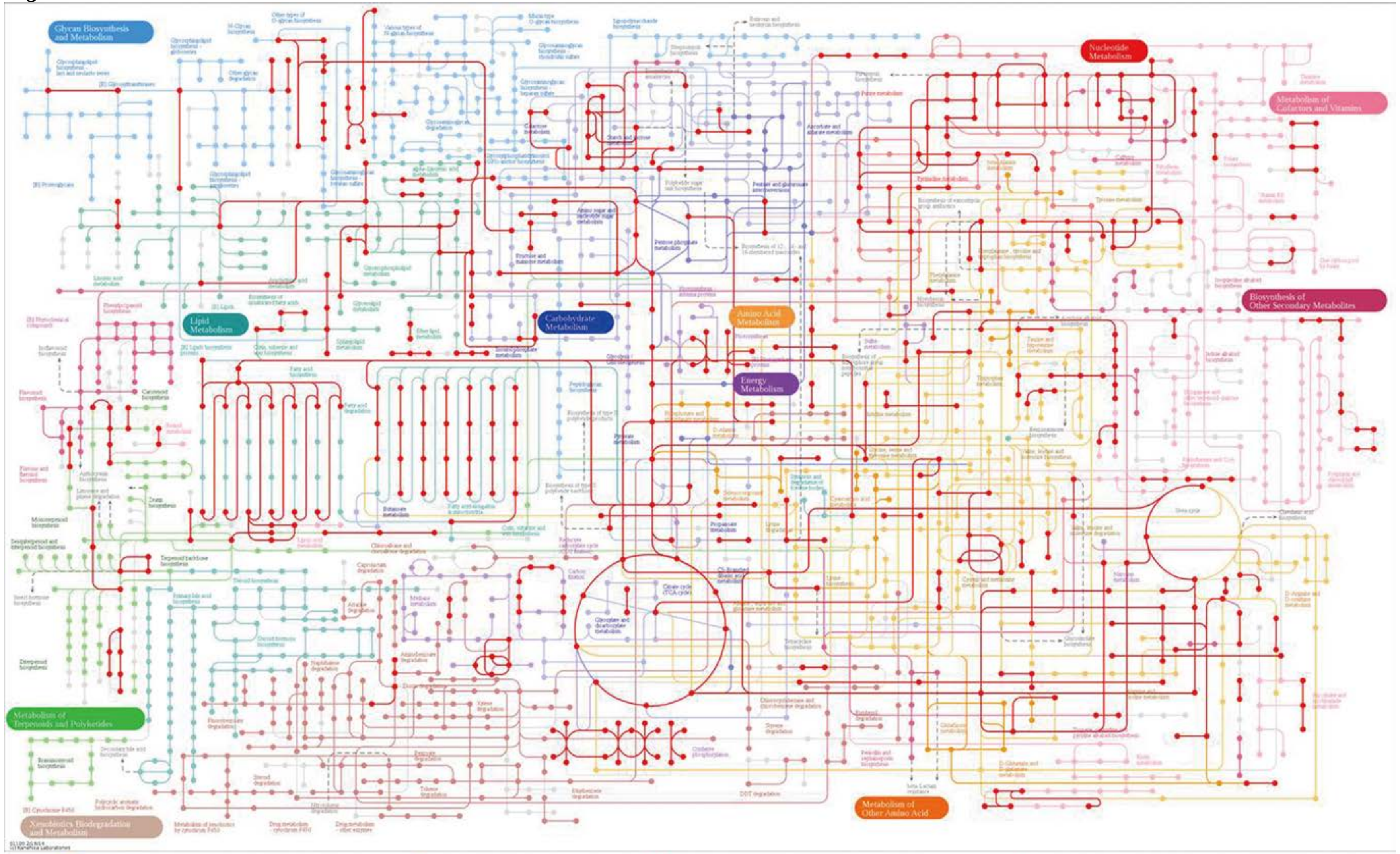

- Glycan Biosynthesis and Metabolism
- Lipid Metabolism
- Metabolism of Terpenoids and Polyketides
- Energy Metabolism
- Carbohydrate Metabolism
- Amino Acid Metabolism
- Metabolism of Cofactors and Vitamins
- Biosynthesis of Other Secondary Metabolites
- Metabolism of Other Amino Acid
- Xenobiotics Biodegradation and Metabolism
- Nucleotide Metabolism

Fig. S6. KEGG pathway analysis of *H. pluvialis* showing differential gene expression (above two fold) between macrozooids (A) and cysts (B). The transcripts expressed at a higher level in cysts were those encoding proteins involved in a wide range of metabolisms.

Fig. S7.

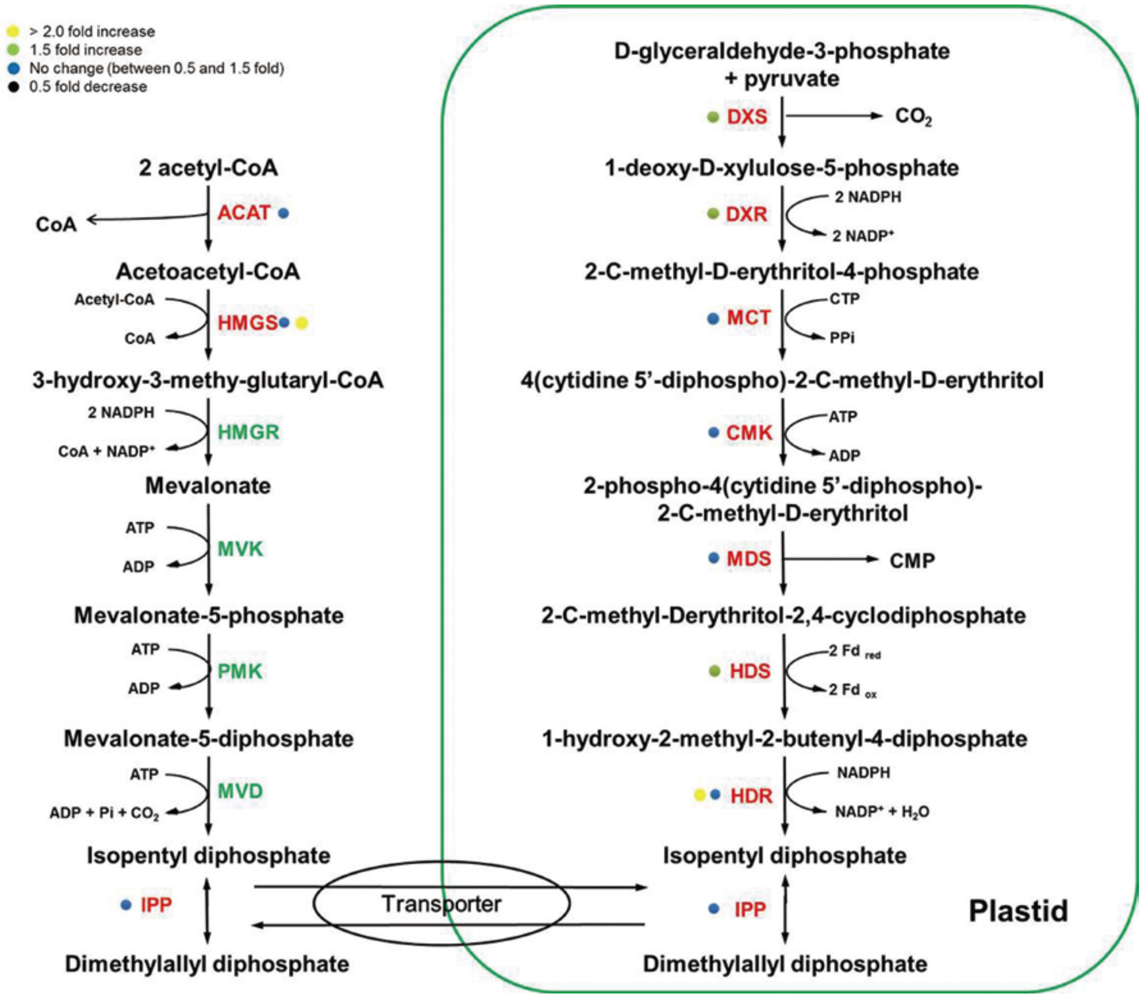

Fig. S7. Mevalonate (MVA) and 2-C-methyl-D-erythritol 4-phosphate (MEP) pathways in *H. pluvialis*. All MEP pathway–related genes were present in *H. pluvialis*. However, only three genes were identified in the mevalonate pathway. The dot color indicates the normalized read number ratio of red (haematocyst) per green (macrozooid) cells, and each dot stands for independent isogroup (gene). ACAT, acetyl-CoA C-acetyltransferase; HMGS, hydroxymethylglutaryl-CoA synthase; HMGR, hydroxymethylglutaryl-CoA reductase; MVK, mevalonate kinase; PMK, phosphomevalonate kinase; MVD, mevalonate dirophosphate decarboxylase; DXS, 1-deoxy-D-xylulose-5-phosphate synthase; DXR, 1-deoxy-D-xylulose 5-phosphate reductoisomerase; MCT, 2-C-methyl-D-erythritol 4-phosphate cytidyltransferase; CMK, 4-diphosphocytidyl-2C-methyl-D-erythritol kinase; MDS, 2-C-methyl-D-erythritol 2,4-cyclodiphosphate synthase; HDS, 1-hydroxy-2-methyl-2-(E)-butenyl-4-diphosphate synthase; HDR, 4-hydroxy-3-methylbut-2-enyl diphosphate reductase; IPP: isopentenyl diphosphate isomerase.

Fig. S8.

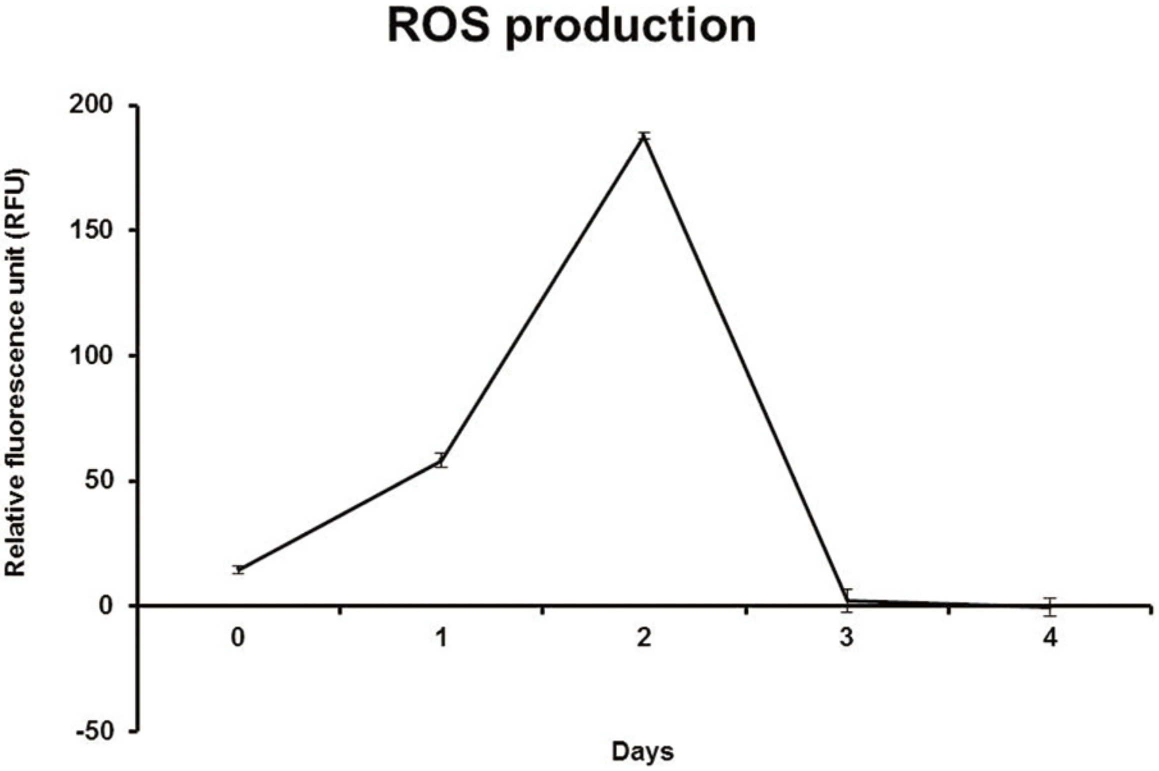

Fig. S8. ROS production of *H. pluvialis*. The late exponential cells of macrozooid were exposed to high light conditions (400 μmol·photon·m^{-2}s^{-1}) for 4 days and the ROS produced from *Haematococcus* cells was measured using H_2DCFDA. The ROS production was expressed by the relative fluorescence unit, which is a fluorescence value normalized by the amounts of cells. Values represent mean ± SD (n=3).
